# The Fungal Frontier: A Comparative Analysis of Methods Used in the Study of the Human Gut Mycobiome

**DOI:** 10.3389/fmicb.2017.01432

**Published:** 2017-07-31

**Authors:** Chloe E. Huseyin, Raul Cabrera Rubio, Orla O’Sullivan, Paul D. Cotter, Pauline D. Scanlan

**Affiliations:** ^1^Teagasc Food Research Centre, Teagasc Fermoy, Ireland; ^2^APC Microbiome Institute, Biosciences Institute, University College Cork Cork, Ireland; ^3^School of Microbiology, University College Cork Cork, Ireland

**Keywords:** mycobiome, gut microbiota, mycobiota, fungi, human, healthy, methodology

## Abstract

The human gut is host to a diverse range of fungal species, collectively referred to as the gut “mycobiome”. The gut mycobiome is emerging as an area of considerable research interest due to the potential roles of these fungi in human health and disease. However, there is no consensus as to what the best or most suitable methodologies available are with respect to characterizing the human gut mycobiome. The aim of this study is to provide a comparative analysis of several previously published mycobiome-specific culture-dependent and -independent methodologies, including choice of culture media, incubation conditions (aerobic versus anaerobic), DNA extraction method, primer set and freezing of fecal samples to assess their relative merits and suitability for gut mycobiome analysis. There was no significant effect of media type or aeration on culture-dependent results. However, freezing was found to have a significant effect on fungal viability, with significantly lower fungal numbers recovered from frozen samples. DNA extraction method had a significant effect on DNA yield and quality. However, freezing and extraction method did not have any impact on either α or β diversity. There was also considerable variation in the ability of different fungal-specific primer sets to generate PCR products for subsequent sequence analysis. Through this investigation two DNA extraction methods and one primer set was identified which facilitated the analysis of the mycobiome for all samples in this study. Ultimately, a diverse range of fungal species were recovered using both approaches, with *Candida* and *Saccharomyces* identified as the most common fungal species recovered using culture-dependent and culture-independent methods, respectively. As has been apparent from ecological surveys of the bacterial fraction of the gut microbiota, the use of different methodologies can also impact on our understanding of gut mycobiome composition and therefore requires careful consideration. Future research into the gut mycobiome needs to adopt a common strategy to minimize potentially confounding effects of methodological choice and to facilitate comparative analysis of datasets.

## Introduction

Our knowledge of the role that the gut microbiota plays in human health and disease has been greatly facilitated by advances in next generation sequencing (NGS) technologies (van Dijk et al., 2014). NGS allows for the detailed investigation of microbial diversity and abundance, enabling researchers to identify specific microbes and microbial populations that are associated with the health or disease status of the host (Iliev et al., 2012; Mar Rodríguez et al., 2015). NGS can also be used by researchers to investigate the influence of host genetics, host environment, and specific intervention strategies (e.g., diet, antibiotic administration, probiotics) on gut microbiota composition (Hoffmann et al., 2013).

To date, the majority of gut microbiome studies have focused on bacteria. However, there is an increasing appreciation that other microbes, such as the fungal fraction of the community (or mycobiome), contribute to host health and well-being (Ott et al., 2008; Chen et al., 2011; Iliev et al., 2012; Mar Rodríguez et al., 2015; Sokol et al., 2016). Fungi are present in the gastrointestinal tracts of humans as commensal organisms (Scanlan and Marchesi, 2008; Hallen-Adams et al., 2015) and transient colonizers (Gouba et al., 2013, 2014; Hoffmann et al., 2013), but also as opportunistic pathogens (Gouba and Drancourt, 2015). Indeed, it has been suggested that the gut mycobiome is an important risk factor in the etiology of a number of diseases, including inflammatory bowel disease (Ott et al., 2008; Iliev et al., 2012; Li et al., 2014; Mukhopadhya et al., 2014; Liguori et al., 2015), obesity (Mar Rodríguez et al., 2015) and chronic hepatitis B infection (Chen et al., 2011). However, it is important to note that, in many studies to date, it has not been clear whether observed differences in gut mycobiome diversity between cases and controls (Chen et al., 2011; Sokol et al., 2016) contribute to disease initiation and progression or are simply a consequence of the disease state. Moreover, the relative impact of other factors (e.g., antibiotic intervention and host genetics) on both the composition and functionality of the gut mycobiome, and/or how they affect the ability of specific fungi to transition from commensalism to pathogenicity (Underhill and Iliev, 2014) remains largely unknown. Therefore, greater research into the fungal fraction of the gut microbiome is required to better understand its potential role in human health and disease.

However, a prerequisite to such research is the establishment of appropriate methodology. This is of crucial importance as microbiome studies have consistently shown that the use of different methodologies can account for considerable variation in the resulting data output (Yu and Morrison, 2004; Bahl et al., 2012; Clooney et al., 2016; Fouhy et al., 2016). Consequently, the choice of method used to study a particular microbiome will greatly impact on the conclusions reached. Although, the impact of method choice on data generated has been demonstrated for numerous studies of the bacterial fraction of the human gut microbiome, there is little information available on the relative merits of different methodologies used to analyze the fungal fraction of this microbial community. In particular, no comparative analysis has been performed to assess the suitability of different DNA extraction methods and PCR primers for fungal specific surveys of the human gut microbiome.

Despite being a relatively new research area (Suhr and Hallen-Adams, 2015; Huseyin et al., 2017) a number of studies on the human gut mycobiome have been published. These studies have used a number of different culture-dependent and -independent methodologies, involving different cultivation media, DNA extraction methods and/or choice of PCR primers to generate amplicons for sequence analysis (Scanlan and Marchesi, 2008; Chen et al., 2011; Hamad et al., 2012; Iliev et al., 2012; Hoffmann et al., 2013; Li et al., 2014; Mukhopadhya et al., 2014; Luan et al., 2015; Mar Rodríguez et al., 2015; Liguori et al., 2015; Sokol et al., 2016). Unfortunately, no critical analysis of these various methodologies has been carried out. Consequently, there is no consensus as to what the most suitable methods for surveying the gut mycobiota are. Moreover, the effect of freezing on samples prior to DNA extraction, which is often the norm following sample collection and, for example, is known to affect the rumen gut mycobiome (Henderson et al., 2013), on human gut mycobiota analysis has not been evaluated. Despite these issues, it is accepted that the fungal ITS region is the most suitable biomarker for NGS-based amplicon sequencing to determine the fungal composition of a microbial community (Bates et al., 2013).

The aim of this study was to compare different culture-dependent and -independent techniques that have been used in previously published studies of the human mycobiome (Yu and Morrison, 2004; Scanlan and Marchesi, 2008; Ghannoum et al., 2010; Iliev et al., 2012) in order to assess their relative merits. More specifically, four different culture media types as well as incubation conditions (aerobic versus anaerobic) were assessed with respect to the recovery of a diverse range of fungal species. Five different DNA extraction methods and eight different fungal PCR primers were also tested with regard to DNA yield and quality, and ability to generate products for sequence analysis, respectively. The impact of a single freeze-thaw cycle and storage at -80°C on both culture-dependent and culture-independent results was also evaluated based on quantitative and qualitative differences observed before and after freezing. Finally, by virtue of the application of NGS to compare the merits of different extraction methods and primer pairs, we also provide insight into the fungal diversity of the healthy human gut mycobiome.

## Materials and Methods

### Experimental Overview

**FIGURE 1:**
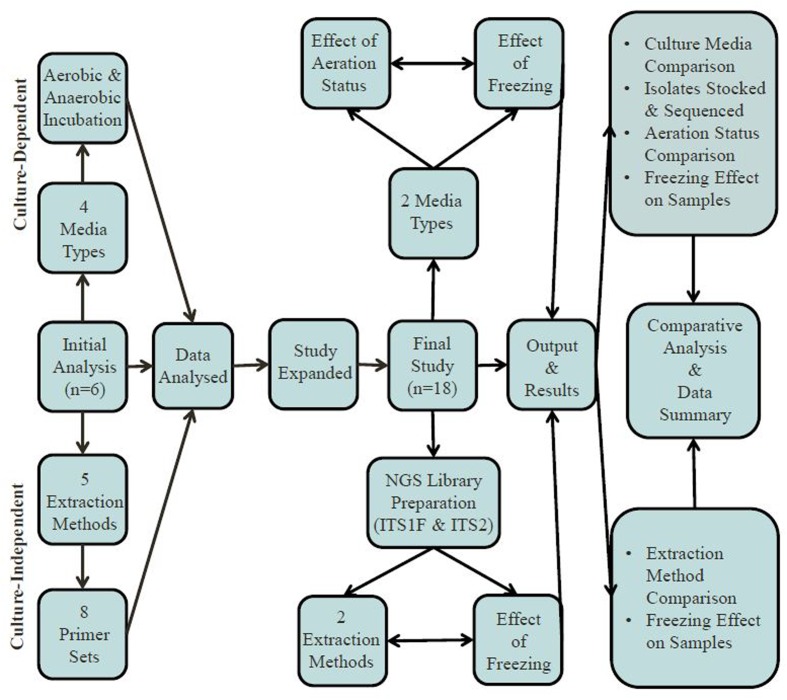
Overview of experimental design.

Fecal samples were collected from eighteen (*n* = 9 male and *n* = 9 female) participants who had not received oral antibiotic or antifungal drugs for at least two months (typically > 6 months) prior to donation; see **Table 1** for subject participant overview. These samples were the focus of both culture-dependent and -independent investigations. Initially, utilizing six fecal samples, the relative impact of four different culture media were assessed with respect to recovery of total fungal numbers and the diversity of fungal species isolated, see Supplementary Information. Two culture media types were selected to assess the effect of aeration status (*n* = 18) and freezing (*n* = 7) of fecal samples on culturable fungi. Culture-independent techniques involved testing and evaluating the effects of five different DNA extraction methods, eight different fungal-specific ITS primer sets and the freezing of fecal samples in the generation of data for gut mycobiome analysis. A summary of the experimental design is given in **Figure 1**. Informed consent was obtained from all subjects in accordance with the Clinical Research Ethics Committee of the Cork Teaching Hospitals (Protocol no. APC022).

**Table 1:** Participant information and selected culture dependent results.

Study participant	Age	Mean fungal counts CFU g^-1^ ±SD	Pure-culture isolate classification (closest relative on database)
F1	41/42	3.83 × 10^3^ ± 5.26 × 10^2^	*Candida albicans*
			*Candida* sp.
			Uncultured *Candida* clone
F2	35	1.25 × 10^2^ ± 1.66 × 10^2^	*Pichia fermentans* / Uncultured compost fungus
F3	29	1.67 × 10^2^ ± 2.15 × 10^2^	*Mucor* sp.
			*Rhodotorula mucilaginosa*
			Uncultured *Candida* clone
			*Mucor circinelloides f. circinelloides*
			*Candida albicans*
			*Epicoccum nigrum*
			Uncultured *Candida* clone
**F4**	46	2.11 × 10^3^ ± 5.05 × 10^2^	^+^*Candida albicans*
F5	30	8.33 × 10^0^ ± 2.89 × 10^1^	*Pseudallescheria boydii*
F6	24	1.36 × 10^3^ ± 4.36 × 10^2^	*Candida albicans*
F7	25	0 ± 0	No isolates
F8	38	1.67 × 10^2^ ± 1.15 × 10^2^	*Candida albicans*
**F9**	35	3.44 × 10^3^ ± 1.93 × 10^3^	*^∗^Aspergillus sojae*
			^+^*Candida parapsilosis*
			^+^*Clavispora lusitaniae*
			*^∗^Penicillium* sp.
			*^∗^Talaromyces diversus*
			*^∗^Talaromyces* sp.
			*^∗^Talaromyces stollii*
			*^∗^Talaromyces variabilis*
			*ˆMeyerozyma caribbica*
M1	39	3.75 × 10^5^± 8.14 × 10^4^	*Candida albicans*
			Uncultured *Saccharomycetales* clone
			*Candida parapsilosis*
			Uncultured *Candida* clone
M2	25	2.9 × 10^3^ ± 8.97 × 10^2^	*Candida albicans*
			*Penicillium* sp.
M3	49	2.63 × 10^3^ ± 8.43 × 10^2^	*Candida albicans*
			*Candida* sp.
			Uncultured *Candida* clone
			*Pichia kudriavzevii*
M4	28	0 ± 0	No isolates
M5	25	8.33 × 10^0^ ± .89 × 10^1^	No sequences passed quality control
M6	39	6.68 × 10^3^ ± 1.07 × 10^3^	*Candida albicans*
			Uncultured *Candida* clone
**M7**	36	6.49 × 10^4^ ± 8.03 × 10^3^	*^∗^Issatchenkia orientalis*
			^+^*Pichia kudriavzevii*
			^+^Uncultured *Pichia* clone
			*ˆCandida parapsilosis*
M8	37	3.33 × 10^1^ ± 4.92 × 10^1^	*Candida albicans*
**M9**	55	8.38 × 10^3^ ± 1.18 × 10^3^	^+^*Candida albicans*
			*^∗^Pichia kudriavzevii*
			*ˆCandida* sp.
			ˆUncultured Saccharomycetales
			*ˆClavispora lusitaniae*


*Study participants in bold text were used for freezing effect on strain recovery. The following symbols denote where strains were recovered from: ^+^Recovered from both fresh and frozen samples, ˆRecovered from frozen samples only, ^∗^Recovered from fresh samples only.*

### Colony PCR and Strain Identification

Colonies were re-streaked onto fresh agar plates to isolate single colonies for stocking of pure cultures and colony PCR. For colony PCR, 200 μl of broth culture was centrifuged at 15,000 rpm for 5 min and the supernatant discarded, 200 μl of sterile 10% sodium dodecyl sulfate was then added to the cell pellet and this was heated to 100°C for 30 min before centrifugation at 15,000 rpm for 10 min. A 1 in 100 dilution of the supernatant was prepared and 2 μl of the dilution was used as a template for a 50 μl PCR. PCR was conducted using the fungal specific primer pair ITS1F and ITS4. PCR conditions were as follows: 25 μL BioMix^TM^ Red, 1 μL each primer (10 pmol concentration), 0.1 μL 20 mg/ml BSA, 0.1 μL DMSO, and 20.8 μl PCR grade dH_2_O). PCR was performed using an Applied Biosystems 2720 Thermal Cycler instrument and PCR conditions consisted of an initial denaturing step for 5 min at 94°C, followed by 35 cycles of 94°C for 30 s, 55°C for 30 s and 72°C for 1 min, with a final extension time of 5 min at 72°C before cooling and holding at 4°C. Where this technique was unsuccessful several subsequent methods (see Supplementary Information) were employed to produce a sufficiently strong PCR product to allow for PCR clean up and Sanger sequencing. PCR products were cleaned up using the QIAquick PCR Purification Kit as per the manufacturer’s instructions. Products were sequenced bi-directionally by Source Bioscience (Waterford, Ireland) and Beckman Coulter Genomics (now Genewiz) (Takeley, United Kingdom). Sequencher software was used in the analysis of the Sanger sequences, where sequences underwent quality control steps such as end trimming and the consensus sequences were created from the forward and reverse reads and identified using BLASTn.

### Freezing Effect Analysis

To assess the impact of freezing on fungal counts and diversity of species recovered we took seven samples that had been stored for at least a month at -80°C [83 ± 53 days (mean ± SD)]. These samples were thawed and treated in the same manner as previously described for the fresh samples. Colonies were stocked from four samples for further strain analysis.

## Culture Independent Methods

### DNA Extraction

Samples from six participants were used to test the effect of five different DNA extraction methods, which had been used previously in mycobiome studies, on total DNA recovery and quality, see **Table 2** for details. These six samples were then stored at -80°C prior to being re-extracted using all five DNA extraction methods.

**Table 2:** Extraction methods used in this study.

Method		Abbreviation used in paper	Reference
QIAamp Fast DNA Stool Mini Kit	As per manufacturer’s instructions	Q	
QIAamp Fast DNA Stool Mini Kit and Bead beating	As per manufacturer’s instructions with addition of 3× 1 min bead beats with 1 min incubation on ice in-between each bead beat.	BB	Scanlan and Marchesi, 2008
QIAamp Fast DNA Stool Mini Kit and Lyticase lysis buffer	Thirty minute incubation using the lysis buffer described in (Iliev et al., 2012) prior to continuation of the extraction using the QIAamp Fast DNA Stool Mini Kit	LYT	Iliev et al., 2012
FastDNA^®^ SPIN Kit	As per manufacturer’s instructions for yeast using buffer CLS-Y.	F	Ghannoum et al., 2010
RBBC (Repeat bead beating + column)	As described in (Yu and Morrison, 2004)	RBBC or R used in some graphs for brevity	Yu and Morrison, 2004


### Culture Dependent Methods

#### Fungal Culturing

Fresh fecal samples were collected and were stored at 4°C until processed (<24 h, typically within 12 h). All samples were serially diluted and spread plated in triplicate onto different media, supplemented with antibiotics (see Supplementary Information, also **Figure 1**). Agar plates were incubated aerobically and anaerobically at 37°C, and counted after 48 h incubation and again at 2 weeks to allow for the detection of potentially slow growing species. Colonies were stocked for each sample based on colony morphology (all unique colony morphologies were picked) with a minimum of three colonies stocked from each donor per media.

Briefly, 200 mg of feces used for each extraction method. Where tubes for bead beating were required they were prepared by adding 250 mg of equal quantities of 0.1 mm and 1.0 zirconium/silica beads and one 2.3 mm zirconium/silica bead. Extracted DNA was quantified and quality checked using the Qubit^®^ 2.0 Fluorometer and associated kits (the Qubit^®^ dsDNA HS Assay Kit, Qubit HS RNA assay kit and Qubit protein assay kit) as well as DNA visualization on stained agarose gels (1% agarose gel stained with SYBR^®^ Safe DNA Gel Stain) and visualized under UV light. Two methods were excluded (F and Q) due to the results obtained from Qubit assays’ and the inability to produce PCR products from DNA extracted using these methods. A further twelve fresh fecal samples were extracted using the remaining three extraction methods (BB, LYT, RBBC). Four samples were also extracted post storage at -80°C using these three extraction methods.

#### PCR and Primer Choice

PCR primers, which have been previously employed to target the fungal ITS region from DNA isolated from a range of different environments (**Table 3**), were used to amplify our extracted DNA.

**Table 3:** Primers used in this study.

Primer pair (forward and reverse)	Sequence forward (5′→3′)	Sequence reverse (5′→3′)	Target region	Ability to generate PCR product (using DNA from 6 participants × 3 methods)	Reference
ITS1F and ITS2	CTTGGTCATTTAGAGGAAGTAA	GCTGCGTTCTTCATCGATGC	ITS1	PCR products from 6/6 BB, 5/6 LYT, and 6/6 RBBC samples	White et al., 1990; Gardes and Bruns, 1993
ITS1F and ITS4	CTTGGTCATTTAGAGGAAGTAA	TCCTCCGCTTATTGATATGC	Entire ITS	PCR products from 1/6 BB, 1/6 LYT, and 3/6 RBBC samples	White et al., 1990; Gardes and Bruns, 1993
BITS and B58S3	ACCTGCGGARGGATCA	GAGATCCRTTGYTRAAAGTT		PCR products from 0/6 BB, 0/6 LYT, and 0/6 RBBC samples	Bokulich and Mills, 2013
ITS5 and ITS2	GGAAGTAAAAGTCGTAACAAGG	GCTGCGTTCTTCATCGATGC	ITS1	PCR products from 3/6 BB, 4/6 LYT, and 3/6 RBBC samples	White et al., 1990
ITS1F _KY01 and ITS2 _KY01	CTHGGTCATTTAGAGGAASTAA	CTRYGTTCTTCATCGDT	ITS1	PCR products from 0/6 BB, 0/6 LYT, and 0/6 RBBC samples	Toju et al., 2012
ITS1F _KY02 and ITS2 _KY02	TAGAGGAAGTAAAAGTCGTAA	TTYRCTRCGTTCTTCATC	ITS1	PCR products from 1/6 BB, 1/6 LYT, and 1/6 RBBC samples	Toju et al., 2012
UNI1 and UNI2	ATGAAGAACGCAGCGAAATGCGATA	GTTGGTTTCTTTTCCTCC	ITS2	PCR products from 4/6 BB, 4/6 LYT, and 2/6 RBBC samples	Heisel et al., 2015
FSEQ and RSEQ	ATGCCTGTTTGAGCGTC	CCTACCTGATTTGAGGTC	ITS2	PCR products from 4/6 BB, 4/6 LYT, and 3/6 RBBC samples	Heisel et al., 2015
ITS1F and ITS2 with Illumina adapters for MiSeq sequencing	TCGTCGGCAGCGTCAGATGTGTATAAGAGACAGCTTGGTCATTTAGAGGAAGTAA	GTCTCGTGGGCTCGGAGATGTGTATAAGAGACAGGCTGCGTTCTTCATCGATGC	ITS1	As for ITS1F and ITS2	


#### Library Preparation

The primer set ITS1F and ITS2 was used for library preparation after modification to contain the Illumina sequencing adapters to allow for sequencing using the Illumina MiSeq. The modified primers were tested against the unmodified primers to assess visually on agarose gels any variation in the amplification of PCR products due to the addition of sequencing adapters.

For library preparation, DNA concentrations were first normalized for the library preparation amplicon PCR reaction, for each of the two chosen extraction methods. Amplicon PCRs were performed in triplicate (DNA 5 μL, BioMix^TM^ Red 50 μL, 5 μL each primer (10 pM concentration), 0.1 μL 10 mg/ml BSA 0.1 μL DMSO and 34.8 μl of PCR grade dH_2_O). PCR was performed using a Applied Biosystems 2720 Thermal Cycler instrument using the following conditions: denaturing for 5 min at 94°C, followed by 35 cycles of 94°C for 30 s, 55°C for 30 s and 72°C for 1 min, with a final extension time of 5 min at 72°C before cooling and holding at 4°C. Each sample replicate was run on an agarose gel as described previously. Sample replicates were then pooled and each pooled sample was then visualized a second time on an agarose gel before being run on an Agilent 2100 Bioanalyzer using the Agilent High Sensitivity DNA Kit as per the manufacturer’s instructions. PCR clean ups were performed as described in the Illumina *16S Metagenomic Sequencing Library Preparation guide* (Illumina, 2014) using Agencourt AMPure XP and cleaned PCR products were checked by on the Agilent 2100 bioanalyzer. Barcodes were added to each sample by index PCR using the Nextera XT Index Kit as described elsewhere (Illumina, 2014). A second clean-up was then performed using Agencourt AMPure XP as described (Illumina, 2014) and samples were quantified using the Qubit 2.0 instrument (Qubit HS dsDNA assay). Samples with a concentration below 2.0 ng/μL were discarded and a second sample aliquot was cleaned post-barcoding and eluted into half of the recommended aliquot of buffer before being re-quantified. Samples were then normalized (to the same concentration) and pooled. Five microliters of each normalized sample were combined and cleaned up using a 1:1 library to Agencourt AMPure XP ratio.

#### Sequencing and Bioinformatics

The libraries were sequenced using the Illumina MiSeq platform according to the Illumina *16S Metagenomic Sequencing Library Preparation* Guide for library denaturing and sample loading. Libraries were loaded at a concentration of 4 nM and PhiX control was added at a 5% concentration. The libraries were clonally amplified directly onto adapters on single-end (SE) flow cells and sequenced with standard Illumina sequencing primers according to manufacturer’s instructions for the using the MiSeq reagent Kit v2 2x250bp.

Sequences were quality checked using PRINSEQ (Schmieder and Edwards, 2011) software, utilizing a q-score above 26 and only including reads above 200 bp in length. The reads were joined with the program fastq-join (Aronesty, 2013), allowing a 20 per cent maximum difference in overlap. Reads were parsed to allow the use of VSEARCH software (Rognes et al., 2016). VSEARCH was used to de-replicate reads, cluster reads into OTU’s and remove chimeric sequences. Taxonomy was assigned to the OTU’s using Qiime against the UNITE database version 7.1 (Kõljalg et al., 2013) and sequences were aligned using MUSCLE (Edgar, 2004). PCoA plots were produced using EMPeror (Vázquez-Baeza et al., 2013).

#### Statistical Analysis

Statistical analyses were performed using GraphPad Prism version 7.00 for Windows, GraphPad Software, La Jolla, CA, United States^1^. We present an α level of 0.05 as a measure of statistical significance, we have denoted levels of significance as shown: ^∗^*p* < 0.05, ^∗∗^*p* < 0.01, ^∗∗∗^*p* < 0.001, and ^∗∗∗∗^*p* < 0.0001. Data was tested for normality and where appropriate either parametric or non-parametric, paired, and unpaired tests were performed.

#### Inclusion of Controls

Controls were included at each step of this study, for culture-dependent methods the diluent used to serially dilute fecal samples was also spread plated and incubated as per the culture media plates used for fecal samples. During the culture-independent analysis extraction controls were included every time DNA extractions were performed, for each extraction method by ‘extracting’ an empty tube and treating it the same as for the tubes containing fecal samples. These extractions were quantified and visualized similarly for the DNA extractions from fecal samples as well as being included in the initial amplicon PCR to assess that no PCR product was produced. Negative (PCR dH_2_O) and positive controls (*Candida albicans* DNA) for PCR were included for all PCR reactions.

## Results

### Culture-Dependent Results

**FIGURE 2:**
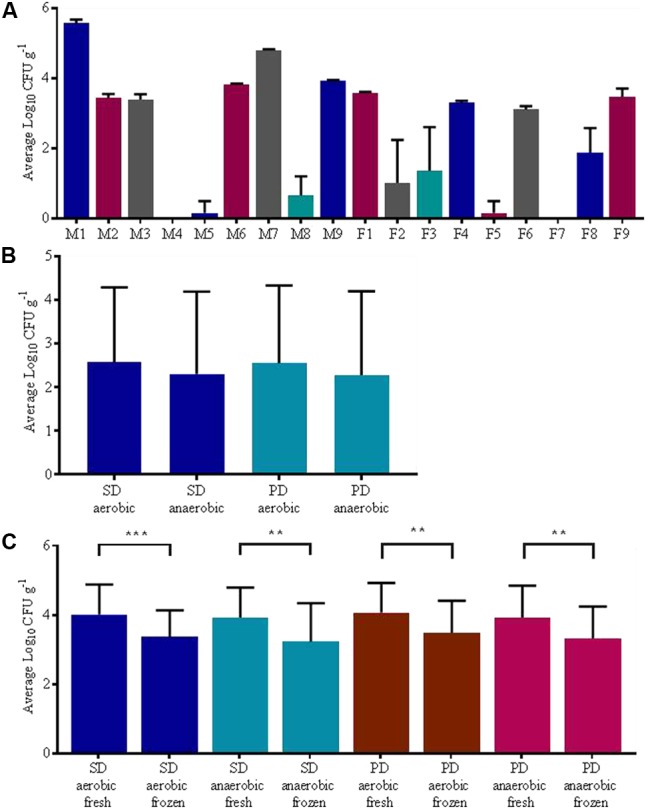
Culture dependent results, mean fungal load ± SD; **(A)** participant, **(B)** effect of aeration status, **(C)** effect of freezing. Abbreviations: Sabouraud dextrose (SD) and potato dextrose (PD); Statistical significance levels ^∗^*p* < 0.05, ^∗∗^*p* < 0.01, ^∗∗∗^*p* < 0.001, and ^∗∗∗∗^*p* < 0.0001.

Significant differences (*p* < 0.0001, repeated measures ANOVA) in fungal load between individuals were apparent. Of the eighteen individuals analyzed, three did not have any culturable fungi and, among the other participants, the average fungal load per individual ranged from 8.33 × 10^0^ ± 2.89 × 10^1^ CFU g^-1^ to 3.75 × 10^5^± 8.14 × 10^4^ CFU g^-1^ (see also **Figure 2A**). We found no significant effect of aeration (i.e., whether plates were cultured aerobically or anaerobically) on fungal counts irrespective of the media type tested (*p* > 0.05 repeated measures ANOVA), see **Figure 2B**. Freezing of samples significantly reduced the recovery of culturable fungi from samples with one freeze thaw cycle resulting in approximately a 10-fold reduction in fungal numbers post freezing compared to fresh samples (*p* < 0.0001, repeated measures ANOVA, adjusted *p* < 0.01 Holm–Sidak’s multiple comparisons test) (**Figure 2C**).

We isolated a number of morphologically different fungal taxa which were subsequently identified using Sanger sequencing of the entire ITS region and have been summarized in **Table 1** and Supplementary Table S1. The identity of the culturable species varied greatly between individuals, with some individuals harboring just one species whereas others were positive for a number of species. The most common genus detected through culture dependent analysis was *Candida*, with 13 individuals’ positive for the genus. Dixon’s media and Czapek-dox media both recovered *Candida albicans* only (see Supplementary Information), whereas a greater variety of fungal species was recovered using Sabouraud dextrose and potato dextrose media. The fungal genera that grew on both Sabouraud and potato dextrose media were very similar and included *Candida* spp. (*C. albicans, C. parapsilosis, and Candida* species), *Issatchenkia orientalis* (*C. krusei*), *Clavispora lusitaniae*, *Pichia kudriavzevii, Penicillium* sp. and *Talaromyces* spp. However, *Mucor* sp. was only isolated using Sabouraud media.

### Culture-Independent Results

**FIGURE 3:**
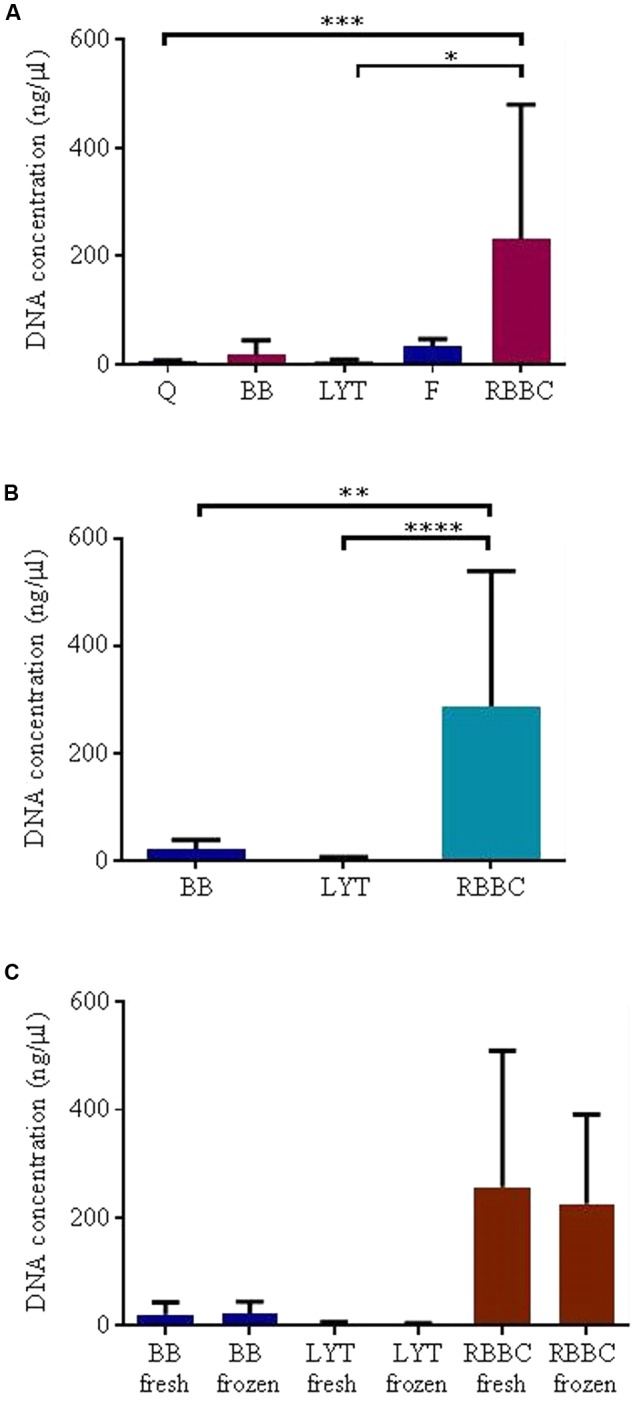
DNA extraction results, mean ± SD; **(A)** five methods, **(B)** three methods, **(C)** freezing. Statistical significance levels ^∗^*p* < 0.05, ^∗∗^*p* < 0.01, ^∗∗∗^*p* < 0.001, and ^∗∗∗∗^*p* < 0.0001.

The choice of DNA extraction method significantly affected total DNA yield. A statistically significant difference in DNA yield was observed between the five extraction methods initially tested (*p* < 0.001, Friedman) thus *post hoc* analysis was performed (adjusted 0.001 < *p* < 0.9999 Dunn’s multiple comparisons test, see **Figure 3A**). It was particularly notable that extraction method F resulted in poor quality DNA that contained high levels of protein (225.67 ± 207.71 μg/ml). The Q extraction method, yielded low DNA concentrations (2.93 ± 2.42 ng/μl, i.e., irrespective of the sample being extracted fresh or post freezing) and samples extracted using this method also resulted in poor amplification of the ITS product (see **Table 3**). Thus, these methods were deemed unsuitable and were excluded from further analysis.

Based on these data the sample size was increased (*n* = 18) to evaluate three of the most promising extraction methods (RBBC, BB and LYT), and noted a significant difference in DNA yield between all three methods (*p* < 0.0001, Friedman, adjusted 0.0001 < *p* < 0.05 Dunn’s multiple comparisons test), see also **Figure 3B**. The average DNA concentration was 21.71 ± 18.3 ng/μl, 3.05 ± 1.95 ng/μl, and 289.49 ± 288.59 ng/μl for the BB, LYT, and RBBC methods, respectively. No significant effect of freezing on DNA extraction for these three methods was observed, see **Figure 3C**.

Each of the primer sets test gave different results with two sets (BITS and B58S3 and ITS1F_KY01 and ITS2_KY01) failing to generate any PCR products for any of the samples tested, see **Table 3**. Based on their relative ability to generate PCR products from the highest number of samples, primer set ITS1F and ITS2 was identified as a suitable primer set to generate culture-independent, sequencing data. It proved difficult to generate amplicons from DNA samples extracted using the LYT method (see **Table 3**) and where amplicons were generated, sequencing of the amplicons failed (see Supplementary Information). Thus, only PCR products generated from DNA extracted using the BB and RBBC extraction methods were sequenced.

**FIGURE 4:**
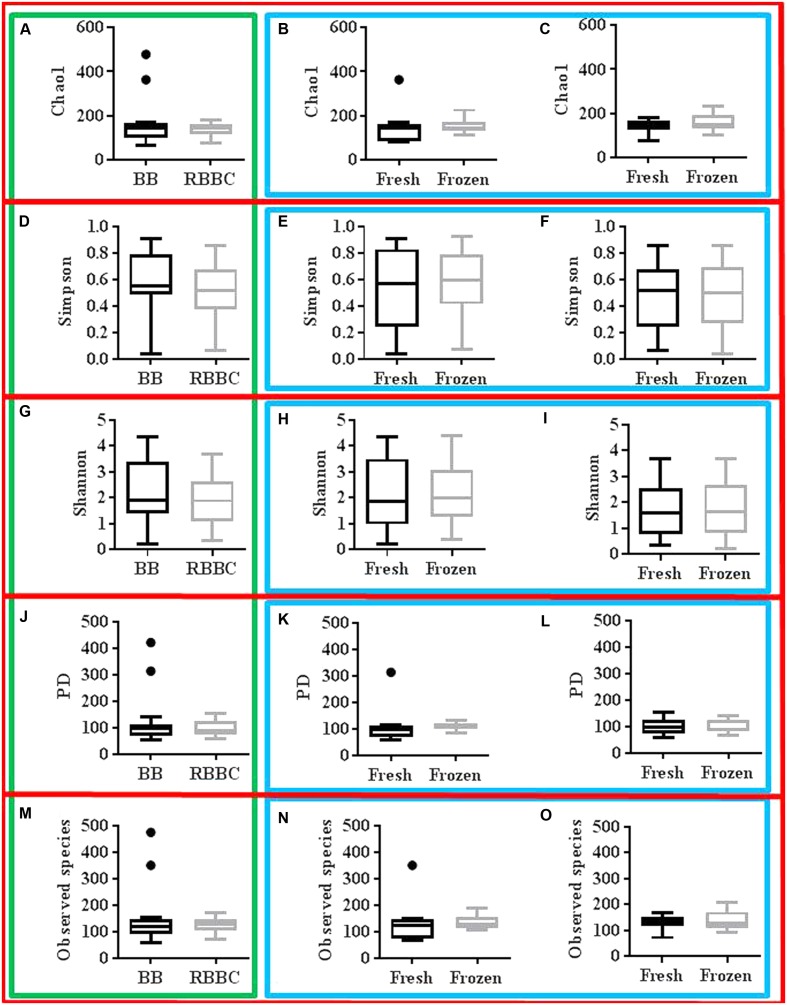
Tuckey graphs of α-diversity measures red: grouping by diversity measure, green: grouping by extraction method charts, blue: grouping by freezing effect charts **(A–C)**: Chao1; **(A)** extraction method, **(B)** freezing effect BB, **(C)** freezing effect RBBC **(D–F)**: Simpson; **(D)** extraction method, **(E)** freezing effect BB, **(F)** freezing effect RBBC **(G–I)**: Shannon; **(G)** extraction method, **(H)** freezing effect BB, **(I)** freezing effect RBBC **(J–L)**: PD; **(J)** extraction method, **(K)** freezing effect BB, **(L)** freezing effect RBBC **(M–O)**: Observed species; **(M)** extraction method, **(N)** freezing effect BB, **(O)** freezing effect RBBC. “●” represents the specific value of the data point, and is denoted in these graphs to show that this datapoint is outside of the 75th percentile + 1.5 the interquartile range.

The sequencing effort yielded 12,408,385 read with an average of 221,578 reads per sample post quality control, which corresponded to 1861 OTU’s. Alpha diversity metrics were assessed for normality using the D’Agostino and Pearson normality test and paired parametric/non-parametric (*t*-test/Wilcoxon matched-pairs signed rank test) statistical tests were performed as appropriate. No significant effect on α-diversity was observed due to extraction method or freezing (for each extraction method) as can be seen in **Figure 4**. The effect of gender on α-diversity metrics was also investigated. Notably, gender significantly affected the Simpson diversity index with women significantly higher than men (*p* = 0.0093 one-way ANOVA, *p* < 0.05 Bonferroni’s multiple comparisons test).

**FIGURE 5:**
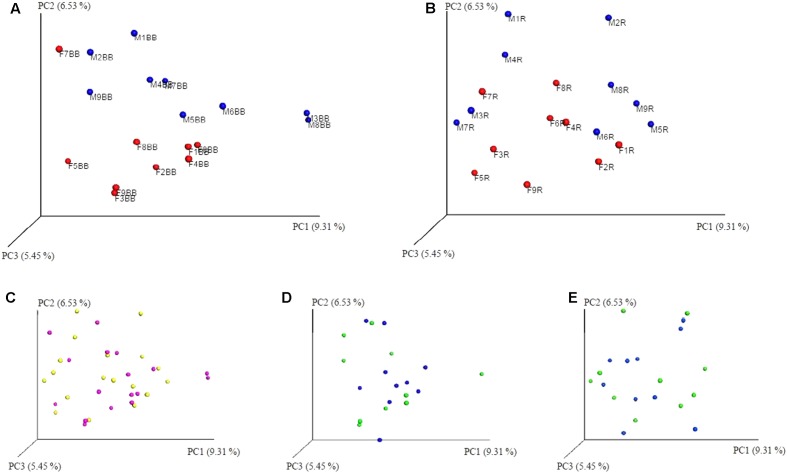
Unweighted PCoA plots **(A)** BB method (red female/blue male); **(B)** RBBC method (red female/blue male); **(C)** BB & RBBC fresh (BB pink/R Yellow); **(D)** BB method (green fresh/blue frozen); **(E)** RBBC method (green fresh/blue frozen). ‘R’ used for chart labelling is an abbreviation for RBBC method.

**FIGURE 6:**
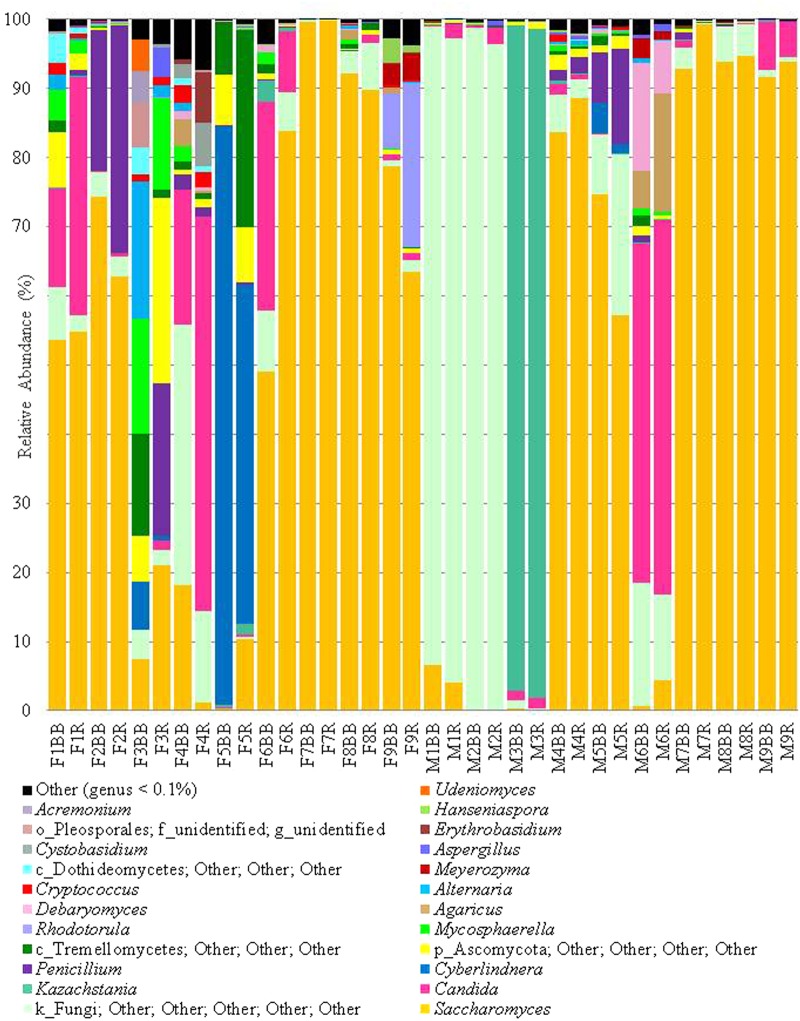
Extraction methods by participant, (genus level), genera at >0.1%.

**FIGURE 7:**
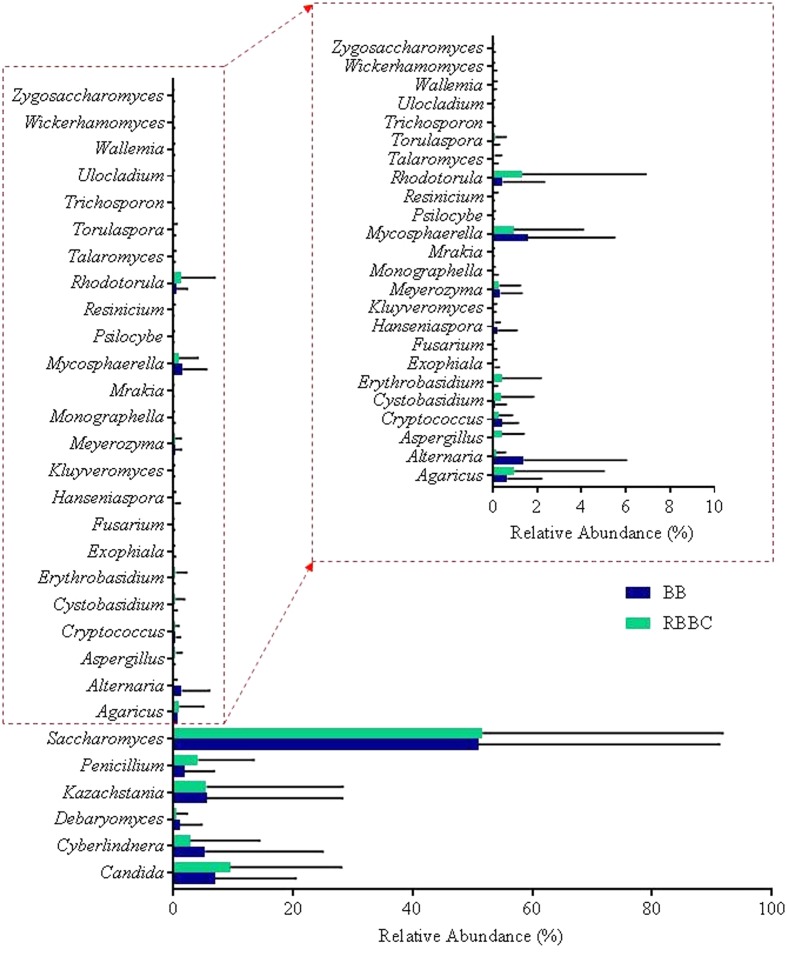
Top 30 fully assigned genera for each extraction method.

In addition to the α-diversity, the effect on β-diversity and taxonomy was also assessed, it was apparent that mycobiome composition was not significantly affected by either extraction method or freezing of samples (Wilcoxon matched-pairs signed rank test, *p* > 0.05; as can be seen in **Figures 5**, **6** and also in Supplementary Figures S1–S3). The most prevalent fungal genera identified in this dataset are *Saccharomyces, Candida, Kazachstania, Cyberlindnera*, and *Penicillium* (see **Figure 7**). Contrary to culture-dependent analysis where only 13 participants were positive for the genus *Candida*, a culture-independent approach indicated that all individuals were in fact positive for *Candida* although for some individuals the percentage is extremely small.

Similarly, a small, yet significant effect of gender was observed at the family and genus levels with respect to β-diversity for samples extracted using the RBBC method (Mann–Whitney, *p* < 0.05; family level *p* = 0.0418, genus level *p* = 0.0342) and some separation can also be observed in the PCoA plots in **Figures 5A,B**. In this dataset, the male participants are generally qualitatively less diverse than the female participants as can be seen in the Unweighted PCoA plots, see **Figures 5A,B** and Supplementary Figure S2, with the exception of the female participant F7 who has a mycobiome dominated >99% by *Saccharomyces* and is more similar to the male participants (**Figure 5A**).

## Discussion

Consistent with studies of the bacterial fraction of the gut microbiome, our data demonstrates that different methodologies can impact on analyses of the composition of the human gut mycobiome as has been observed elsewhere (Suhr et al., 2016). A number of different culture media and conditions were investigated in this study and showed that overall, total fungal numbers (i.e., fungal counts) were only significantly affected by freezing and not by aeration status and media type. Freezing also had a qualitative effect on the diversity of fungal species recovered, with a number of fungal species only recovered from fresh fecal samples, see **Table 1**. In our initial assessment of media choice, we did observe a reduced diversity of fungal species recovered using Dixon’s media and Czapek-dox media compared to Sabouraud dextrose and potato dextrose media during the initial analysis. Subsequently these two media were dropped from further analysis (see Supplementary Information). However, we did not observe any difference in diversity of fungal species recovered when comparing Sabouraud dextrose and potato dextrose media, with the exception of fungal genus *Mucor* which was only detected using Sabouraud dextrose media. However, an important caveat of our culture-dependent approach is that although all unique colony-types (i.e., any colony with a unique morphology) were sampled, stocked and analyzed, morphology can mask genetic variation which could potentially affect the diversity of species reported.

The ability of different DNA extraction methods and PCR primer sets to generate high quality, clean DNA for PCR, as well as PCR products for downstream sequencing applications, varied significantly. Of the five DNA extraction methods that were specifically chosen based on a comprehensive survey of the literature (Yu and Morrison, 2004; Scanlan and Marchesi, 2008; Ghannoum et al., 2010; Iliev et al., 2012), see **Table 2**, only two were employed for ITS sequence data generation and subsequent downstream analysis (BB and RBBC). These methods were selected based on their ability to extract a sufficiently high DNA yield that was of high quality, free from PCR inhibitors, and shown to be suitable for the production of ITS amplicons (see **Table 3**). We postulated that although the RBBC method gave the highest DNA yield that this did not necessarily mean that DNA obtained using other extraction methods that give significantly lower yields would provide qualitatively different results. Based on this rationale PCR products generated using both RBBC and BB methodologies were sequenced. Moreover, these two methods are frequently used in many recent microbiome studies and, thus, can potentially provide the added bonus of allowing for comparative analysis of the mycobiota across a range of studies as well as facilitating a retrospective analysis of the mycobiome using stored DNA samples that were extracted using these approaches.

A number of the most commonly used primer sets, that target the ITS region, were selected for this study. Note, this is not an exhaustive list of all primers sets available for mycobiome analysis, however our choices were based on rationale outlined earlier (Hawksworth et al., 2011). Moreover, fungal ITS databases provide the most comprehensive collection of fungal data for use in sequence based studies of the mycobiomes (Santamaria et al., 2012). Our results show that different ITS-specific primers can vary greatly in their ability to produce products for downstream analysis even from the same sample. Based on our analysis, the use of some primer sets, e.g., BITS and B58S3, might erroneously indicate that no fungal species are present in a particular sample(s) due to a lack of PCR product. However, the primer set we ultimately selected from the primer sets tested (ITS1F and ITS2) was successfully used for library preparation and could not only amplify ITS products from our samples (see **Table 3**), but also gave products of a size suitable for sequence analysis. Given that our primary prerequisite for primer choice in this study was to identify a primer set that could generate products from all samples, and all except this one primer set were unable to produce products for a number of samples (see **Table 3**), we specifically chose to sequence amplicons generated with primer set ITS1F and ITS2 only. Although our final primer choice generated multiple sized products as has also been reported in the literature by others (Tang et al., 2015), we found this did not adversely affect sequencing. With respect to the choice of sequencing platform, a comparative analysis of different NGS platforms has already been performed using mock fungal communities and shown that Illumina MiSeq platform outperforms others for mycobiome analysis (Tang et al., 2015), hence this platform was employed for this study.

Our experimental design and analysis allowed us to investigate variation in the gut mycobiome of individuals and across culture-dependent and -independent methods. Using culture-independent analysis, we recovered a minimum of seven different fungal genera per individual and as many as ten and eleven genera for individuals that did not have cultivable fungal species (F7 and M4, respectively; **Table 3**). Whilst a number of individuals sequence profiles were dominated by a single genus, it is evident that many individuals were host to a diversity of fungi (**Figure 6**). It was noted that *C. albicans* was the most frequently isolated species using culture-dependent methodologies whereas *Saccharomyces* was the dominant fungal genus detected using culture-independent techniques. In fact, *Saccharomyces* was the most abundant genus for several individuals (F2, F6, F7, F8, F9, M7, M8, and M9) despite not being detected using culture-dependent techniques. There are a number of possible explanations for this observation, including our colony sampling strategy, a greater ability of *Candida* to grow on the culture media chosen, or perhaps the high prevalence of *Saccharomyces* is in fact dietary contamination, which has also been postulated by others (Hoffmann et al., 2013). The latter theory is consistent with the fact that *Saccharomyces cerevisiae* has an optimal growth temperature range of between 25 and 35°C (Pizarro et al., 2008) and therefore has most often been isolated at the following temperatures: 25°C (Agırbaslı et al., 2005), 27°C (Strati et al., 2016), and 30°C (Chen et al., 2011). The genus *Penicillium* was also detected at high levels using the culture-independent approach for a number of individuals (F2, F3, and M5) but was not detected in samples from these individuals tested by culturing. In this case, perhaps the specific *Penicillium* strains in question were not viable. *Penicillum* was isolated by culturing from two other individuals in this study (F9 and M2) using the same methodologies despite only representing <1% of the mycobiome composition of these individuals when assessed from a culture-independent perspective. Similarly, *Pichia* spp. were detected by culturing in samples from F2, M3, M7 and M9, but were only detected by both culture-dependent and -independent methods for M7. Dietary contamination was explicitly evident in some individuals. For example, sequences related to the fungal genus *Agaricus* were detected for M6, this fungal genus consists of edible species such as *Agaricus bisporus*, i.e. ‘button mushrooms’. Similarly, the genus *Kazachstania* which dominates the mycobiome of M3 contains species that have been reported in fermented drinks such as kefir (Marsh et al., 2013) and kombucha (Marsh et al., 2014) and therefore the presence of this fungi may also be attributed to diet.

Nonetheless, culture-independent analysis indicates that the diversity of the gut mycobiome varies greatly between individuals (see **Figure 6**), a finding that has been previously observed in other studies of the healthy gut (Scanlan and Marchesi, 2008; Hoffmann et al., 2013; Heisel et al., 2015; Strati et al., 2016; Suhr et al., 2016). Another finding that is consistent with other mycobiome studies is the presence of fungi in the human gut for which we know very little about in terms of both taxonomic affiliation and functionality, and also their interactions with and ability to influence human health in the host. For example, many sequences could not be assigned any meaningful taxonomic level; this is of particular note for participants M1 and M2, whom despite having a high culturable fungal load which included *Candida* spp., over 90% of reads from these individuals could not be assigned below kingdom level and indeed sequences from all individuals contained the descriptor “Fungi; Other; Other; Other; Other; Other” (**Figure 7**), albeit to varying extents. Moreover, culture-dependent techniques resulted in the isolation of between zero and six genera per individual, whereas culture-independent reveal a minimum of seven genera per individual at an abundance greater than 0.1%. These findings highlight the urgent need for the development of novel media to target the currently “unculturable” fungal members of the gut microbiota. Access to this fraction of the gut microbiome is required if we are to further expand our understanding of both the diversity and role of the human gut mycobiome (Huseyin et al., 2017) that has been revealed by NGS. This expansion was highlighted in 2013 with respect to the UNITE database (Kõljalg et al., 2013) where an approximate 5-fold increase in the number of sequences deposited between from 2005 to 2013 was reported. These deposited sequences are grouped into a number of hypothesized fungal species to accompany fungal reference sequences in the database and considerable differences between the number of available reference sequences and the number of hypothesized species are evident for each fungal phylum, this can limit the ability to bioinformatically assign meaningful taxonomic affiliation to sequence data. As has been observed in this study, and is evident in the literature (Huseyin et al., 2017), the composition of the human gut mycobiome is dominated by two phyla, namely, Ascomycota and Basidiomycota. The results presented by Kõljalg and colleagues in 2013 report 20,754 hypothesized species belonging to the Ascomycota phylum, yet only 287 available reference sequences; and 20,804 hypothesized species from Basidiomycota with 1,476 available reference sequences. Although the numbers of hypothesized species and reference sequences for Ascomycota and Basidiomycota have increased considerably since 2013, with UNITE version 7.2 (updated 8th June 2017) reporting 3,310 and 3,484 reference sequences for each phylum, respectively, the number of species hypothesized to exist is still considerably greater (33,051 and 28,743, respectively) (Kõljalg et al., 2013). Without continued efforts to generate additional reference genomes, and the curation of fungal sequences already in the databases, or indeed the production of new targeted databases (Tang et al., 2015), issues in assigning complete taxonomic information during culture-independent analyses is likely to continue and imbalances between the number of hypothesized species and number of reference sequences available for fungi will ultimately limit our understanding of these communities.

Finally, we observed a small but significant effect of gender for the quantitative sequence data generated from the RBBC method. However, this effect is likely driven by slight differences in the abundances of the most prevalent families and genera in this dataset that was not observed in the BB method. Although our study does indicate an effect of gender, it is important to note that this study was not primarily designed for the analysis of the mycobiome with respect to gender and our gender specific analyses were of a retrospective nature. However, any potential confounding effect from gender is important not only in human studies but also, for example, in murine or animal studies as often only one gender is used for analysis (Iliev et al., 2012). It will be interesting to see if future analyses utilizing larger cohorts of individuals will also observe this emerging trend in compositional differences due to gender as we and others have reported (Strati et al., 2016).

## Conclusion

As research into the gut mycobiome is still in its infancy, it is imperative that we adopt and apply a consensus methodology. Our data shows that both the RBBC and BB DNA extraction methods are efficient extraction methods that provide clean, high quality and amplifiable DNA for fungal PCRs. However, given the significantly higher DNA yield evident for the RBBC method over the BB method we recommend the use of the RBBC DNA extraction for gut mycobiome analysis. Moreover, consistent use of primers such as the ITS1F and ITS2 pair, selected for use in this study based on their superior ability to amplify fungal products of a suitable size for sequence analysis for the entire sample-set compared to other primers tested is also recommended. Appropriate DNA extraction method and primer choice, together with careful consideration of how samples are processed and stored will allow future research to provide novel insight into the gut mycobiome across groups of interest as well as providing the capacity to compare and contrast findings across multiple studies.

## Availability of Data

The sample datasets supporting the conclusions of this article are available in the European Nucleotide Archive repository, http://www.ebi.ac.uk/ena under the following accession number: PRJEB20103.

## Author Contributions

CH, PC, and PS designed the experiments. CH performed all of the experiments and performed statistical analysis of the data. RR developed the ITS bioinformatics pipeline and performed the bioinformatics analysis of the data, OO’S provided bioinformatics assistance. CH and PS wrote the manuscript and RR and PC contributed to the final preparation of the manuscript. All authors approved the final manuscript.

## Conflict of Interest Statement

The authors declare that the research was conducted in the absence of any commercial or financial relationships that could be construed as a potential conflict of interest.

## Footnotes

1 www.graphpad.com
