# Supplementary material for: The Fungal Frontier: A Comparative Analysis of Methods Used in the Study of the Human Gut Mycobiome
Source: Front Microbiol. 2017 Jul 31;8:1432. doi: 10.3389/fmicb.2017.01432 (PMC5534473; doi:10.3389/fmicb.2017.01432)
Supplement: Supplementary file 1 [file Table_1.DOCX]

Supplementary Material

The fungal frontier: A comparative analysis of methods used in the study of the human gut mycobiome

Chloe E. Huseyin^1, 2, 3^, Raul Cabrera Rubio^1, 2^, Orla O’Sullivan^1, 2^, Paul D. Cotter^1, 2*^, Pauline D. Scanlan^2*^

*** Correspondence:** Corresponding Authors: Pauline D. Scanlan: paulinescanlan@yahoo.co.uk or p.scanlan@ucc.ie Paul D. Cotter: paul.cotter@teagasc.ie

## Supplementary Table 1.

Fungal species recovered from different individuals using different media

| **Isolate number** | **Subject ID** | **Nearest blast hit** | **Query coverage %** | **Identity %** | **Culture media** | **Aerobic status** | **Freezing status** |
| --- | --- | --- | --- | --- | --- | --- | --- |
| H1B1 | F4 | *Candida albicans* strain | 99 | 99 | Czapek-dox | Aerobic | Fresh |
| H1B2 | F4 | *Candida albicans* strain | 98 | 96 | Dixons | Aerobic | Fresh |
| H1B3 | F4 | *Candida albicans* strain | 99 | 99 | Potato dextrose | Aerobic | Fresh |
| H1B4 | F4 | *Candida albicans* strain | 99 | 99 | Potato dextrose | Aerobic | Fresh |
| H1B5 | F4 | *Candida albicans* strain | 100 | 100 | Sabouraud dextrose | Aerobic | Fresh |
| H1B6 | F4 | *Candida albicans* strain | 100 | 100 | Sabouraud dextrose | Aerobic | Fresh |
| H1B7 | F4 | *Candida albicans* strain | 100 | 99 | Czapek-dox | Anaerobic | Fresh |
| H1B8 | F4 | *Candida albicans* strain | 99 | 99 | Dixons | Anaerobic | Fresh |
| H1B9 | F4 | *Candida albicans* strain | 100 | 100 | Potato dextrose | Anaerobic | Fresh |
| H1B10 | F4 | *Candida albicans* strain | 100 | 100 | Sabouraud dextrose | Anaerobic | Fresh |
| H1B11 | F1 | *Candida* sp. | 99 | 99 | Potato dextrose | Aerobic | Fresh |
| H1B12 | F1 | Uncultured *Candida* clone | 100 | 100 | Potato dextrose | Anaerobic | Fresh |
| H1B13 | F1 | *Candida albicans* isolate | 100 | 100 | Sabouraud dextrose | Aerobic | Fresh |
| H1B14 | F1 | Uncultured *Candida* clone | 100 | 100 | Sabouraud dextrose | Anaerobic | Fresh |
| H1B15 | F2 | Uncultured compost fungus*/Pichia fermentans* | 100 | 99 | Potato dextrose | Aerobic | Fresh |
| H1B17 | F3 | *Rhodotorula mucilaginosa* strain | 100 | 100 | Potato dextrose | Aerobic | Fresh |
| H1B18 | F3 | Uncultured *Candida* clone | 100 | 100 | Sabouraud dextrose | Aerobic | Fresh |
| H1B19 | F3 | *Mucor* sp. | 98 | 89 | Sabouraud dextrose | Aerobic | Fresh |
| H1B20 | F3 | *Mucor circinelloides f. circinelloides* strain | 97 | 99 | Sabouraud dextrose | Aerobic | Fresh |
| H1B21 | F3 | *Candida albicans* isolate | 100 | 99 | Czapek-dox | Anaerobic | Fresh |
| H1B22 | F3 | *Candida albicans* isolate | 100 | 100 | Dixons | Anaerobic | Fresh |
| H1B23 | F3 | *Candida albicans* isolate | 100 | 100 | Sabouraud dextrose | Anaerobic | Fresh |
| H1B24 | F3 | *Candida albicans* strain | 100 | 100 | Potato dextrose | Aerobic | Fresh |
| H1B25 | F3 | *Epicoccum nigrum* | 98 | 99 | Sabouraud dextrose | Aerobic | Fresh |
| H1B26 | F3 | Uncultured *Candida* clone | 100 | 100 | Sabouraud dextrose | Anaerobic | Fresh |
| H1B27 | F4 | *Candida albicans* strain | 99 | 99 | Potato dextrose | Aerobic | Fresh |
| H1B28 | F4 | *Candida albicans* strain | 100 | 100 | Potato dextrose | Aerobic | Fresh |
| H1B29 | F4 | *Candida albicans* strain | 99 | 99 | Potato dextrose | Aerobic | Fresh |
| H1B30 | F4 | *Candida albicans* strain | 100 | 99 | Potato dextrose | Anaerobic | Fresh |
| H1B31 | F4 | *Candida albicans* isolate | 100 | 99 | Potato dextrose | Anaerobic | Fresh |
| H1B32 | F4 | *Candida albicans* strain | 100 | 100 | Sabouraud dextrose | Aerobic | Fresh |
| H1B33 | F4 | *Candida albicans* strain | 99 | 99 | Sabouraud dextrose | Aerobic | Fresh |
| H1B34 | F4 | *Candida albicans* strain | 100 | 100 | Sabouraud dextrose | Aerobic | Fresh |
| H1B35 | F4 | *Candida albicans* strain | 99 | 100 | Sabouraud dextrose | Anaerobic | Fresh |
| H1B36 | F4 | *Candida albicans* strain | 99 | 99 | Sabouraud dextrose | Anaerobic | Fresh |
| H1B37 | F4 | *Candida albicans* strain | 99 | 99 | Sabouraud dextrose | Anaerobic | Fresh |
| H1B38 | F5 | *Pseudallescheria boydii* strain | 100 | 99 | Sabouraud dextrose | Aerobic | Fresh |
| H1B39 | F6 | *Candida albicans* isolate | 100 | 100 | Potato dextrose | Aerobic | Fresh |
| H1B40 | F6 | *Candida albicans* isolate | 100 | 99 | Potato dextrose | Aerobic | Fresh |
| H1B41 | F6 | *Candida albicans* isolate | 100 | 100 | Potato dextrose | Aerobic | Fresh |
| H1B42 | F6 | *Candida albicans* isolate | 99 | 96 | Potato dextrose | Anaerobic | Fresh |
| H1B43 | F6 | *Candida albicans* isolate | 100 | 99 | Potato dextrose | Anaerobic | Fresh |
| H1B44 | F6 | *Candida albicans* isolate | 99 | 100 | Potato dextrose | Anaerobic | Fresh |
| H1B45 | F6 | *Candida albicans* isolate | 100 | 100 | Sabouraud dextrose | Aerobic | Fresh |
| H1B46 | F6 | *Candida albicans* isolate | 99 | 99 | Sabouraud dextrose | Aerobic | Fresh |
| H1B47 | F6 | *Candida albicans* isolate | 100 | 100 | Sabouraud dextrose | Aerobic | Fresh |
| H1B48 | F6 | *Candida albicans* isolate | 99 | 99 | Sabouraud dextrose | Anaerobic | Fresh |
| H1B49 | F6 | Uncultured *Candida* clone | 100 | 99 | Sabouraud dextrose | Anaerobic | Fresh |
| H1B50 | F6 | *Candida albicans* isolate | 99 | 99 | Sabouraud dextrose | Anaerobic | Fresh |
| H1B51 | F8 | *Candida albicans* isolate | 100 | 100 | Potato dextrose | Aerobic | Fresh |
| H1B52 | F8 | *Candida albicans* isolate | 100 | 100 | Potato dextrose | Aerobic | Fresh |
| H1B53 | F8 | *Candida albicans* isolate | 100 | 100 | Potato dextrose | Aerobic | Fresh |
| H1B54 | F8 | *Candida albicans* isolate | 100 | 99 | Potato dextrose | Anaerobic | Fresh |
| H1B55 | F8 | *Candida albicans* isolate | 99 | 100 | Potato dextrose | Anaerobic | Fresh |
| H1B56 | F8 | *Candida albicans* isolate | 100 | 100 | Potato dextrose | Anaerobic | Fresh |
| H1B57 | F8 | *Candida albicans* isolate | 99 | 99 | Potato dextrose | Anaerobic | Fresh |
| H1B58 | F8 | *Candida albicans* isolate | 100 | 100 | Potato dextrose | Anaerobic | Fresh |
| H1B59 | F8 | *Candida albicans* strain | 100 | 99 | Potato dextrose | Anaerobic | Fresh |
| H1B60 | F8 | *Candida albicans* isolate | 99 | 99 | Potato dextrose | Anaerobic | Fresh |
| H1B61 | F8 | *Candida albicans* isolate | 100 | 100 | Potato dextrose | Anaerobic | Fresh |
| H1B62 | F8 | *Candida albicans* isolate | 100 | 100 | Sabouraud dextrose | Aerobic | Fresh |
| H1B63 | F8 | *Candida albicans* isolate | 100 | 100 | Sabouraud dextrose | Aerobic | Fresh |
| H1B64 | F8 | *Candida albicans* isolate | 100 | 100 | Sabouraud dextrose | Aerobic | Fresh |
| H1B65 | F8 | *Candida albicans* isolate | 99 | 100 | Sabouraud dextrose | Anaerobic | Fresh |
| H1B66 | F8 | *Candida albicans* isolate | 99 | 99 | Sabouraud dextrose | Anaerobic | Fresh |
| H1B67 | F9 | *Aspergillus sojae* strain | 38 | 85 | Potato dextrose | Aerobic | Fresh |
| H1B68 | F9 | *Talaromyces variabilis* strain | 91 | 79 | Potato dextrose | Aerobic | Fresh |
| H1B69 | F9 | *Talaromyces* sp | 91 | 98 | Potato dextrose | Aerobic | Fresh |
| H1B70 | F9 | *Talaromyces stollii* | 100 | 100 | Potato dextrose | Aerobic | Fresh |
| H1B71 | F9 | *Candida parapsilosis* isolate | 100 | 100 | Potato dextrose | Aerobic | Fresh |
| H1B72 | F9 | *Talaromyces stollii* | 100 | 99 | Potato dextrose | Aerobic | Fresh |
| H1B73 | F9 | *Candida parapsilosis* isolate | 100 | 100 | Potato dextrose | Aerobic | Fresh |
| H1B74 | F9 | *Candida parapsilosis* isolate | 100 | 100 | Potato dextrose | Aerobic | Fresh |
| H1B75 | F9 | *Clavispora lusitaniae* strain | 99 | 98 | Potato dextrose | Anaerobic | Fresh |
| H1B76 | F9 | *Candida parapsilosis* isolate | 99 | 99 | Potato dextrose | Anaerobic | Fresh |
| H1B77 | F9 | *Candida parapsilosis* isolate | 100 | 97 | Potato dextrose | Anaerobic | Fresh |
| H1B78 | F9 | *Candida parapsilosis* isolate | 100 | 99 | Potato dextrose | Anaerobic | Fresh |
| H1B79 | F9 | *Candida parapsilosis* isolate | 100 | 99 | Potato dextrose | Anaerobic | Fresh |
| H1B80 | F9 | *Candida parapsilosis* isolate | 100 | 100 | Potato dextrose | Anaerobic | Fresh |
| H1B81 | F9 | *Candida parapsilosis* isolate | 100 | 99 | Potato dextrose | Anaerobic | Fresh |
| H1B82 | F9 | *Candida parapsilosis* isolate | 100 | 100 | Potato dextrose | Anaerobic | Fresh |
| H1B83 | F9 | *Penicillium* sp. | 100 | 99 | Sabouraud dextrose | Aerobic | Fresh |
| H1B84 | F9 | *Candida parapsilosis* isolate | 100 | 100 | Sabouraud dextrose | Aerobic | Fresh |
| H1B85 | F9 | *Talaromyces diversus* isolate | 79 | 100 | Sabouraud dextrose | Aerobic | Fresh |
| H1B86 | F9 | *Talaromyces diversus* isolate | 72 | 100 | Sabouraud dextrose | Aerobic | Fresh |
| H1B87 | F9 | *Talaromyces diversus* isolate | 72 | 100 | Sabouraud dextrose | Aerobic | Fresh |
| H1B88 | F9 | *Candida parapsilosis* isolate | 99 | 100 | Sabouraud dextrose | Aerobic | Fresh |
| H1B89 | F9 | *Candida parapsilosis* isolate | 100 | 100 | Sabouraud dextrose | Aerobic | Fresh |
| H1B90 | F9 | *Talaromyces diversus* strain | 84 | 99 | Sabouraud dextrose | Aerobic | Fresh |
| H1B91 | F9 | *Talaromyces diversus* strain | 87 | 99 | Sabouraud dextrose | Aerobic | Fresh |
| H1B92 | F9 | *Candida parapsilosis* isolate | 100 | 100 | Sabouraud dextrose | Aerobic | Fresh |
| H1B93 | F9 | *Candida parapsilosis* isolate | 99 | 100 | Sabouraud dextrose | Aerobic | Fresh |
| H1B94 | F9 | *Clavispora lusitaniae* strain | 98 | 98 | Sabouraud dextrose | Aerobic | Fresh |
| H1B95 | F9 | *Clavispora lusitaniae* strain | 100 | 98 | Sabouraud dextrose | Anaerobic | Fresh |
| H1B96 | F9 | *Candida parapsilosis* isolate | 99 | 99 | Sabouraud dextrose | Anaerobic | Fresh |
| H1B97 | F9 | *Clavispora lusitaniae* isolate | 98 | 99 | Sabouraud dextrose | Anaerobic | Fresh |
| H1B98 | F9 | *Candida parapsilosis* isolate | 100 | 99 | Sabouraud dextrose | Anaerobic | Fresh |
| H1B99 | F9 | *Candida parapsilosis* isolate | 100 | 99 | Sabouraud dextrose | Anaerobic | Fresh |
| H1B100 | M1 | *Candida albicans* isolate | 100 | 99 | Czapek-dox | Aerobic | Fresh |
| H1B101 | M1 | Uncultured *Saccharomycetales* clone | 97 | 98 | Czapek-dox | Aerobic | Fresh |
| H1B102 | M1 | *Candida albicans* isolate | 98 | 99 | Dixons | Aerobic | Fresh |
| H1B103 | M1 | Uncultured *Candida* clone | 99 | 100 | Dixons | Aerobic | Fresh |
| H1B104 | M1 | *Candida parapsilosis* isolate | 99 | 99 | Potato dextrose | Aerobic | Fresh |
| H1B105 | M1 | Uncultured *Candida* clone | 100 | 100 | Potato dextrose | Aerobic | Fresh |
| H1B106 | M1 | Uncultured *Candida* clone | 100 | 99 | Sabouraud dextrose | Aerobic | Fresh |
| H1B107 | M1 | Uncultured *Candida* clone | 100 | 99 | Dixons | Anaerobic | Fresh |
| H1B108 | M1 | *Candida albicans* isolate | 100 | 100 | Potato dextrose | Aerobic | Fresh |
| H1B109 | M1 | *Candida albicans* isolate | 100 | 100 | Potato dextrose | Aerobic | Fresh |
| H1B110 | M2 | *Candida albicans* strain | 100 | 99 | Czapek-dox | Aerobic | Fresh |
| H1B111 | M2 | *Candida albicans* strain | 100 | 99 | Czapek-dox | Aerobic | Fresh |
| H1B112 | M2 | *Candida albicans* strain | 99 | 99 | Dixons | Aerobic | Fresh |
| H1B113 | M2 | *Candida albicans* strain | 100 | 100 | Dixons | Aerobic | Fresh |
| H1B114 | M2 | *Candida albicans* strain | 99 | 99 | Potato dextrose | Aerobic | Fresh |
| H1B115 | M2 | *Candida albicans* strain | 100 | 99 | Potato dextrose | Aerobic | Fresh |
| H1B116 | M2 | *Candida albicans* strain | 99 | 99 | Sabouraud dextrose | Aerobic | Fresh |
| H1B117 | M2 | *Candida albicans* strain | 100 | 99 | Czapek-dox | Anaerobic | Fresh |
| H1B118 | M2 | *Candida albicans* strain | 100 | 99 | Dixons | Anaerobic | Fresh |
| H1B119 | M2 | *Candida albicans* strain | 100 | 100 | Potato dextrose | Anaerobic | Fresh |
| H1B120 | M2 | *Candida albicans* strain | 100 | 100 | Sabouraud dextrose | Anaerobic | Fresh |
| H1B121 | M2 | *Candida albicans* strain | 100 | 100 | Potato dextrose | Aerobic | Fresh |
| H1B122 | M2 | *Penicillium* sp. | 100 | 98 | Potato dextrose | Aerobic | Fresh |
| H1B123 | M2 | *Candida albicans* strain | 99 | 100 | Potato dextrose | Anaerobic | Fresh |
| H1B124 | M2 | *Candida albicans* strain | 100 | 99 | Sabouraud dextrose | Aerobic | Fresh |
| H1B125 | M2 | *Candida albicans* strain | 99 | 99 | Sabouraud dextrose | Anaerobic | Fresh |
| H1B126 | M3 | *Candida albicans* isolate | 100 | 99 | Czapek-dox | Aerobic | Fresh |
| H1B127 | M3 | *Candida albicans* isolate | 100 | 99 | Czapek-dox | Aerobic | Fresh |
| H1B128 | M3 | Uncultured *Candida* clone | 100 | 99 | Dixons | Aerobic | Fresh |
| H1B129 | M3 | *Candida albicans* isolate | 99 | 100 | Dixons | Aerobic | Fresh |
| H1B130 | M3 | *Candida* sp. | 100 | 99 | Potato dextrose | Aerobic | Fresh |
| H1B131 | M3 | Uncultured *Candida* clone | 100 | 99 | Potato dextrose | Aerobic | Fresh |
| H1B132 | M3 | Uncultured *Candida* clone | 100 | 99 | Sabouraud dextrose | Aerobic | Fresh |
| H1B133 | M3 | *Candida albicans* isolate | 100 | 100 | Sabouraud dextrose | Aerobic | Fresh |
| H1B134 | M3 | *Candida albicans* strain | 100 | 99 | Czapek-dox | Anaerobic | Fresh |
| H1B135 | M3 | Uncultured *Candida* clone | 100 | 99 | Dixons | Anaerobic | Fresh |
| H1B136 | M3 | *Candida albicans* isolate | 100 | 100 | Potato dextrose | Anaerobic | Fresh |
| H1B137 | M3 | *Candida albicans* isolate | 100 | 100 | Sabouraud dextrose | Anaerobic | Fresh |
| H1B138 | M3 | *Pichia kudriavzevii* strain | 99 | 99 | Potato dextrose | Aerobic | Fresh |
| H1B139 | M3 | Uncultured *Candida* clone | 100 | 100 | Potato dextrose | Anaerobic | Fresh |
| H1B140 | M3 | *Candida albicans* isolate | 100 | 100 | Sabouraud dextrose | Aerobic | Fresh |
| H1B141 | M3 | Uncultured *Candida* clone | 100 | 100 | Sabouraud dextrose | Anaerobic | Fresh |
| H1B142 | M6 | *Candida albicans* isolate | 100 | 100 | Potato dextrose | Aerobic | Fresh |
| H1B143 | M6 | *Candida albicans* isolate | 99 | 100 | Potato dextrose | Aerobic | Fresh |
| H1B144 | M6 | Uncultured *Candida* clone | 100 | 99 | Potato dextrose | Aerobic | Fresh |
| H1B145 | M6 | *Candida albicans* isolate | 100 | 100 | Potato dextrose | Anaerobic | Fresh |
| H1B146 | M6 | *Candida albicans* isolate | 100 | 100 | Potato dextrose | Anaerobic | Fresh |
| H1B147 | M6 | Uncultured *Candida* clone | 100 | 99 | Potato dextrose | Anaerobic | Fresh |
| H1B148 | M6 | *Candida albicans* isolate | 100 | 100 | Sabouraud dextrose | Aerobic | Fresh |
| H1B149 | M6 | *Candida albicans* isolate | 99 | 100 | Sabouraud dextrose | Aerobic | Fresh |
| H1B150 | M6 | *Candida albicans* isolate | 100 | 100 | Sabouraud dextrose | Aerobic | Fresh |
| H1B151 | M6 | *Candida albicans* isolate | 100 | 100 | Sabouraud dextrose | Anaerobic | Fresh |
| H1B152 | M6 | *Candida albicans* isolate | 100 | 100 | Sabouraud dextrose | Anaerobic | Fresh |
| H1B153 | M6 | *Candida albicans* isolate | 100 | 100 | Sabouraud dextrose | Anaerobic | Fresh |
| H1B154 | M7 | *Issatchenkia orientalis* isolate | 99 | 100 | Potato dextrose | Aerobic | Fresh |
| H1B155 | M7 | *Pichia kudriavzevii* | 99 | 99 | Potato dextrose | Aerobic | Fresh |
| H1B156 | M7 | Uncultured *Pichia* clone | 99 | 99 | Potato dextrose | Aerobic | Fresh |
| H1B157 | M7 | *Issatchenkia orientalis* isolate | 100 | 100 | Potato dextrose | Anaerobic | Fresh |
| H1B158 | M7 | *Pichia kudriavzevii* | 100 | 99 | Potato dextrose | Anaerobic | Fresh |
| H1B159 | M7 | Uncultured *Pichia* clone | 99 | 99 | Potato dextrose | Anaerobic | Fresh |
| H1B160 | M7 | *Pichia kudriavzevii*strain | 99 | 96 | Sabouraud dextrose | Aerobic | Fresh |
| H1B161 | M7 | *Issatchenkia orientalis* isolate | 99 | 100 | Sabouraud dextrose | Aerobic | Fresh |
| H1B162 | M7 | *Pichia kudriavzevii*strain | 99 | 99 | Sabouraud dextrose | Aerobic | Fresh |
| H1B163 | M7 | Uncultured *Pichia* clone | 99 | 99 | Sabouraud dextrose | Anaerobic | Fresh |
| H1B164 | M7 | Uncultured *Pichia* clone | 99 | 99 | Sabouraud dextrose | Anaerobic | Fresh |
| H1B165 | M7 | Uncultured *Pichia* clone | 99 | 95 | Sabouraud dextrose | Anaerobic | Fresh |
| H1B166 | M8 | *Candida albicans* isolate | 100 | 100 | Potato dextrose | Aerobic | Fresh |
| H1B167 | M8 | *Candida albicans* isolate | 100 | 100 | Potato dextrose | Anaerobic | Fresh |
| H1B168 | M8 | *Candida albicans* isolate | 100 | 99 | Sabouraud dextrose | Aerobic | Fresh |
| H1B169 | M8 | *Candida albicans* isolate | 100 | 100 | Sabouraud dextrose | Anaerobic | Fresh |
| H1B170 | M8 | *Candida albicans* isolate | 100 | 100 | Sabouraud dextrose | Anaerobic | Fresh |
| H1B171 | M9 | *Candida albicans* strain | 100 | 100 | Potato dextrose | Aerobic | Fresh |
| H1B172 | M9 | *Candida albicans* strain | 100 | 100 | Potato dextrose | Aerobic | Fresh |
| H1B173 | M9 | *Candida albicans* strain | 100 | 100 | Potato dextrose | Aerobic | Fresh |
| H1B174 | M9 | *Candida albicans* strain | 99 | 100 | Potato dextrose | Anaerobic | Fresh |
| H1B175 | M9 | *Candida albicans* strain | 100 | 99 | Potato dextrose | Anaerobic | Fresh |
| H1B176 | M9 | *Candida albicans* strain | 100 | 99 | Potato dextrose | Anaerobic | Fresh |
| H1B177 | M9 | *Candida albicans* strain | 100 | 100 | Sabouraud dextrose | Aerobic | Fresh |
| H1B178 | M9 | *Candida albicans* strain | 100 | 100 | Sabouraud dextrose | Aerobic | Fresh |
| H1B179 | M9 | *Pichia kudriavzevii* strain | 99 | 100 | Sabouraud dextrose | Aerobic | Fresh |
| H1B180 | M9 | *Candida albicans* strain | 100 | 100 | Sabouraud dextrose | Aerobic | Fresh |
| H1B181 | M9 | *Candida albicans* strain | 100 | 100 | Sabouraud dextrose | Anaerobic | Fresh |
| H1B182 | M9 | *Candida albicans* strain | 100 | 100 | Sabouraud dextrose | Anaerobic | Fresh |
| H1B183 | M9 | *Candida albicans* strain | 100 | 100 | Sabouraud dextrose | Anaerobic | Fresh |
| H1B184 | F4 | *Candida albicans* strain | 99 | 99 | Potato dextrose | Aerobic | Frozen |
| H1B185 | F4 | *Candida albicans* strain | 99 | 99 | Potato dextrose | Aerobic | Frozen |
| H1B186 | F4 | *Candida albicans* strain | 99 | 100 | Potato dextrose | Aerobic | Frozen |
| H1B187 | F4 | *Candida albicans* strain | 100 | 99 | Potato dextrose | Anaerobic | Frozen |
| H1B188 | F4 | *Candida albicans* strain | 100 | 99 | Potato dextrose | Anaerobic | Frozen |
| H1B189 | F4 | *Candida albicans* strain | 99 | 100 | Potato dextrose | Anaerobic | Frozen |
| H1B190 | F4 | *Candida albicans* strain | 100 | 100 | Sabouraud dextrose | Aerobic | Frozen |
| H1B191 | F4 | *Candida albicans* strain | 100 | 100 | Sabouraud dextrose | Aerobic | Frozen |
| H1B192 | F4 | *Candida albicans* strain | 99 | 99 | Sabouraud dextrose | Aerobic | Frozen |
| H1B193 | F4 | *Candida albicans* strain | 100 | 89 | Sabouraud dextrose | Anaerobic | Frozen |
| H1B194 | F4 | *Candida albicans* strain | 99 | 99 | Sabouraud dextrose | Anaerobic | Frozen |
| H1B195 | F4 | *Candida albicans* strain | 99 | 99 | Sabouraud dextrose | Anaerobic | Frozen |
| H1B196 | F9 | *Meyerozyma caribbica* strain | 100 | 99 | Potato dextrose | Aerobic | Frozen |
| H1B197 | F9 | *Clavispora lusitaniae* strain | 100 | 96 | Potato dextrose | Aerobic | Frozen |
| H1B198 | F9 | *Candida parapsilosis* isolate | 99 | 100 | Potato dextrose | Aerobic | Frozen |
| H1B199 | F9 | *Clavispora lusitaniae* strain | 87 | 78 | Potato dextrose | Aerobic | Frozen |
| H1B200 | F9 | *Clavispora lusitaniae* strain | 98 | 94 | Potato dextrose | Aerobic | Frozen |
| H1B201 | F9 | *Clavispora lusitaniae* strain | 100 | 99 | Potato dextrose | Anaerobic | Frozen |
| H1B202 | F9 | *Clavispora lusitaniae* strain | 100 | 98 | Potato dextrose | Anaerobic | Frozen |
| H1B203 | F9 | *Candida parapsilosis* isolate | 100 | 100 | Sabouraud dextrose | Aerobic | Frozen |
| H1B204 | F9 | *Candida parapsilosis* isolate | 99 | 100 | Sabouraud dextrose | Aerobic | Frozen |
| H1B205 | F9 | *Candida parapsilosis* isolate | 99 | 100 | Sabouraud dextrose | Aerobic | Frozen |
| H1B206 | F9 | *Clavispora lusitaniae* isolate | 100 | 96 | Sabouraud dextrose | Anaerobic | Frozen |
| H1B207 | F9 | *Clavispora lusitaniae* strain | 100 | 96 | Sabouraud dextrose | Anaerobic | Frozen |
| H1B209 | F9 | *Clavispora lusitaniae* isolate | 100 | 99 | Sabouraud dextrose | Anaerobic | Frozen |
| H1B210 | M7 | Uncultured *Pichia* clone | 99 | 99 | Potato dextrose | Aerobic | Frozen |
| H1B211 | M7 | *Candida parapsilosis* | 99 | 100 | Potato dextrose | Aerobic | Frozen |
| H1B212 | M7 | *Candida parapsilosis* | 100 | 99 | Potato dextrose | Aerobic | Frozen |
| H1B213 | M7 | Uncultured *Pichia* clone | 99 | 99 | Potato dextrose | Anaerobic | Frozen |
| H1B214 | M7 | *Pichia kudriavzevii* strain | 100 | 99 | Potato dextrose | Anaerobic | Frozen |
| H1B215 | M7 | Uncultured *Pichia* clone | 99 | 99 | Potato dextrose | Anaerobic | Frozen |
| H1B216 | M7 | Uncultured *Pichia* clone | 99 | 99 | Sabouraud dextrose | Aerobic | Frozen |
| H1B217 | M7 | Uncultured *Pichia* clone | 99 | 99 | Sabouraud dextrose | Aerobic | Frozen |
| H1B218 | M7 | *Candida parapsilosis* isolate | 100 | 100 | Sabouraud dextrose | Aerobic | Frozen |
| H1B219 | M7 | *Pichia kudriavzevii* strain | 100 | 99 | Sabouraud dextrose | Anaerobic | Frozen |
| H1B220 | M7 | Uncultured *Pichia* clone | 99 | 99 | Sabouraud dextrose | Anaerobic | Frozen |
| H1B221 | M7 | *Pichia kudriavzevii* | 100 | 90 | Sabouraud dextrose | Anaerobic | Frozen |
| H1B223 | M7 | *Pichia kudriavzevii* strain | 100 | 99 | Sabouraud dextrose | Anaerobic | Frozen |
| H1B224 | M9 | *Candida albicans* strain | 99 | 99 | Potato dextrose | Aerobic | Frozen |
| H1B225 | M9 | *Candida albicans* strain | 99 | 97 | Potato dextrose | Aerobic | Frozen |
| H1B226 | M9 | *Candida albicans* strain | 99 | 100 | Potato dextrose | Aerobic | Frozen |
| H1B227 | M9 | *Candida albicans* strain | 100 | 99 | Potato dextrose | Anaerobic | Frozen |
| H1B228 | M9 | Uncultured *Saccharomycetales* clone | 64 | 89 | Potato dextrose | Anaerobic | Frozen |
| H1B230 | M9 | *Clavispora lusitaniae* strain | 100 | 98 | Potato dextrose | Anaerobic | Frozen |
| H1B231 | M9 | *Clavispora lusitaniae* strain | 100 | 98 | Potato dextrose | Anaerobic | Frozen |
| H1B232 | M9 | *Candida albicans* strain | 100 | 100 | Sabouraud dextrose | Aerobic | Frozen |
| H1B233 | M9 | *Candida albicans* strain | 99 | 99 | Sabouraud dextrose | Aerobic | Frozen |
| H1B234 | M9 | *Candida albicans* strain | 99 | 99 | Sabouraud dextrose | Aerobic | Frozen |
| H1B235 | M9 | *Candida albicans* strain | 99 | 100 | Sabouraud dextrose | Aerobic | Frozen |
| H1B236 | M9 | *Candida* sp. | 99 | 98 | Sabouraud dextrose | Anaerobic | Frozen |
| H1B237 | M9 | *Candida albicans* strain | 100 | 99 | Sabouraud dextrose | Anaerobic | Frozen |
