# Supplementary material for: The Fungal Frontier: A Comparative Analysis of Methods Used in the Study of the Human Gut Mycobiome
Source: Front Microbiol. 2017 Jul 31;8:1432. doi: 10.3389/fmicb.2017.01432 (PMC5534473; doi:10.3389/fmicb.2017.01432)
Supplement: Supplementary file 2 [file Data_Sheet_1.DOCX]

Supplementary Material

The fungal frontier: A comparative analysis of methods used in the study of the human gut mycobiome

Chloe E. Huseyin^1, 2, 3^, Raul Cabrera Rubio^1, 2^, Orla O’Sullivan^1, 2^, Paul D. Cotter^1, 2*^, Pauline D. Scanlan^2*^

*** Correspondence:** Corresponding Authors: Pauline D. Scanlan: paulinescanlan@yahoo.co.uk or p.scanlan@ucc.ie Paul D. Cotter: paul.cotter@teagasc.ie

# Supplementary Data

## **Culture-dependent media selection:**

Dixons agar (50µg/ml chloramphenicol and 200 µg/ml cycloheximide) and Czapek-dox agar (50µg/ml chloramphenicol and 100µg/ml gentamycin) were employed alongside Sabouraud dextrose and potato dextrose agar (40µg/ml Chloramphenicol and 50µg/ml Kanamycin) for n=6 samples. However only *C. albicans* was isolated on these Dixons and Czapek-dox media types and no significant effect was observed on count data for any media type either aerobically (repeated measures one-way ANOVA, p = 0.2948) or anaerobically (repeated measures one-way ANOVA, p = 0.2527) thus the Dixon’s media and Czapek dox media were discontinued in our analysis.

## Alternative strategies for the production of a PCR product for colony PCR’s on isolates

- Weak PCR products were used as a template for a subsequent 50µl (nested) PCR.
- The supernatant was diluted further to a 1 in 200 dilution and 2µl was used as the PCR template 50µl PCR.
- The cell pellet was microwaved at 900W for 30 seconds diluted in 500µl of PCR grade water 2µl was used as the PCR template 50µl PCR.
- The annealing temperature was reduced to 53°C during the PCR reaction.
- Greater volumes of BSA and DMSO were used (0.2µl in place of 0.1µl) in the PCR reaction.
- A 10% solution of Chelex 100 was prepared, 10µl of broth culture was inoculated into 90µl of 10% Chelex 100, this was heated for 5 minutes at 95°C, 2µl was then used as a PCR template (50µl PCR).
- Kapa robust was used in place of BioMix red (BSA and DMSO) were omitted.

## LYT method

This method was originally meant to be sequenced giving a total of three extraction methods for our analyses however it was later removed from analysis due to issues with sequencing the library. The samples for this method were prepped similarly to the BB and RBBC methods detailed in the text however during sequencing we believe a carryover of some of the extraction reagents caused our library to fail during sequencing (sequencing attempted and failed twice) and thus we abandoned this method and re-pooled our library excluding these samples. We did not further investigate this, however once these samples were removed from the pools the library sequencing experienced no further issues.
