# Supplementary figures and images for: The Fungal Frontier: A Comparative Analysis of Methods Used in the Study of the Human Gut Mycobiome
Source: Front Microbiol. 2017 Jul 31;8:1432. doi: 10.3389/fmicb.2017.01432 (PMC5534473; doi:10.3389/fmicb.2017.01432)

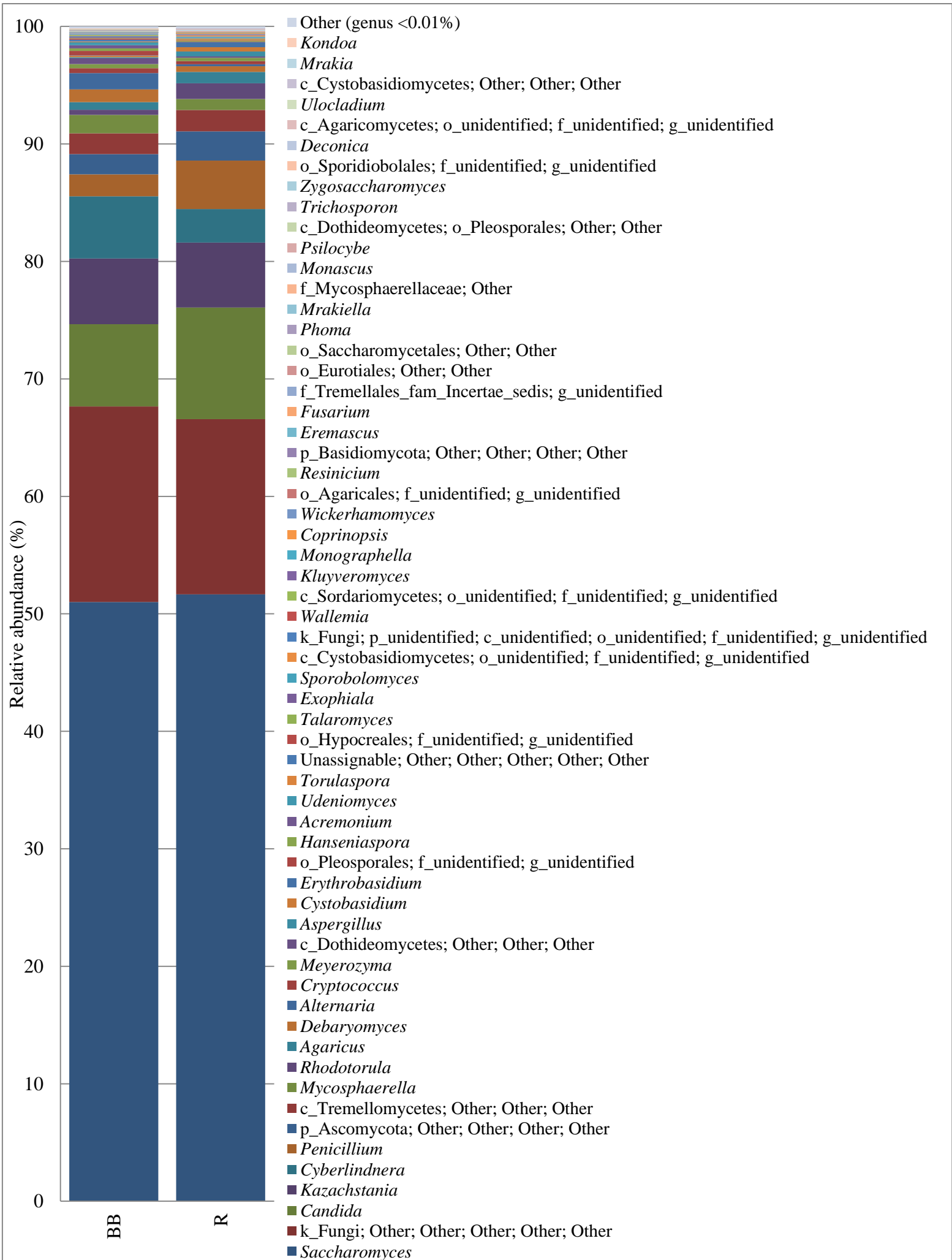

Extraction methods overview, (genus level), genera at >0.01%

Supplement: Supplementary file 3 [file Image_1.PDF]

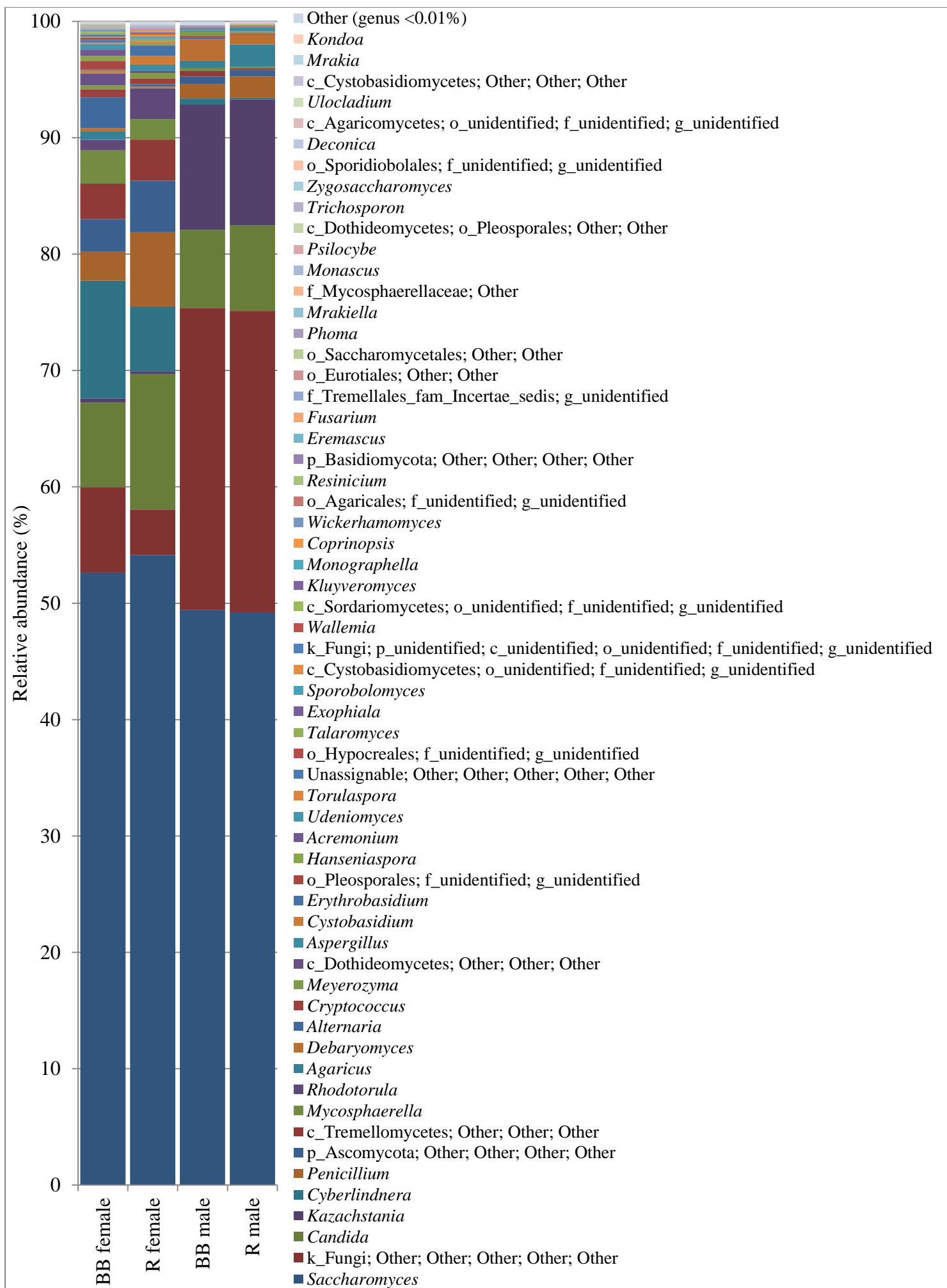

Extraction methods overview split by gender (genus level), genera at >0.01%

Supplement: Supplementary file 4 [file Image_2.PDF]

(A)

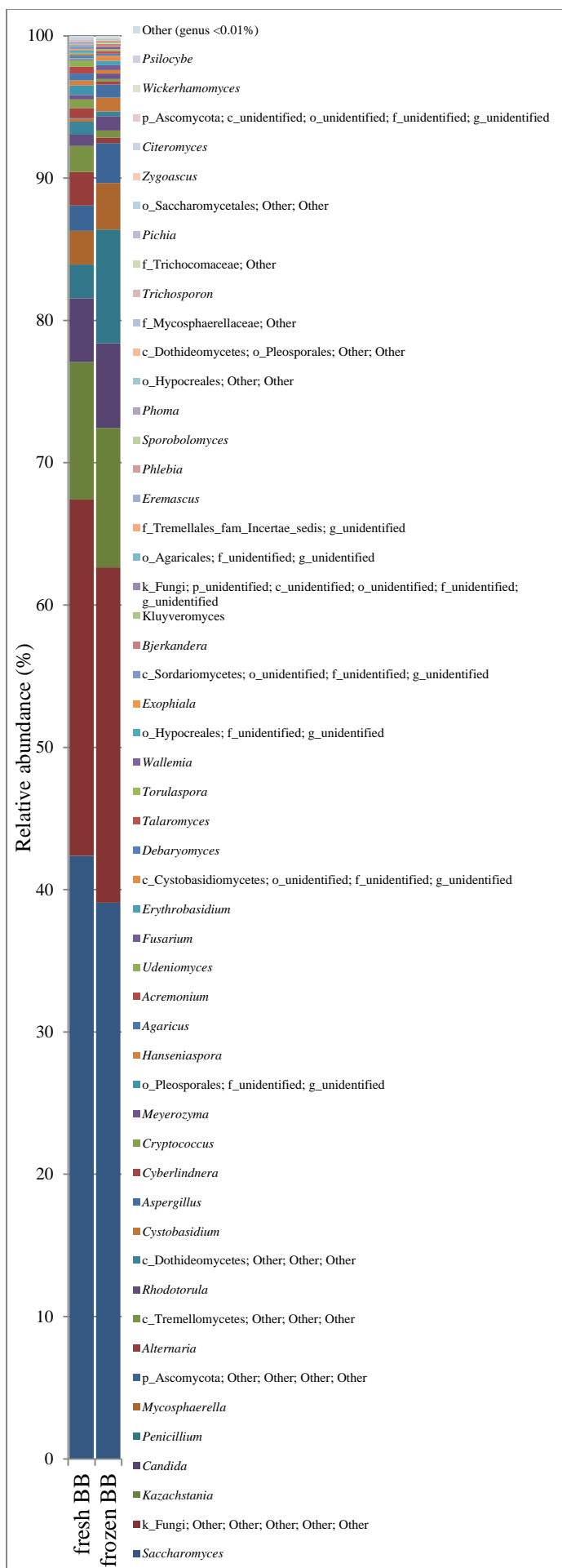

(B)

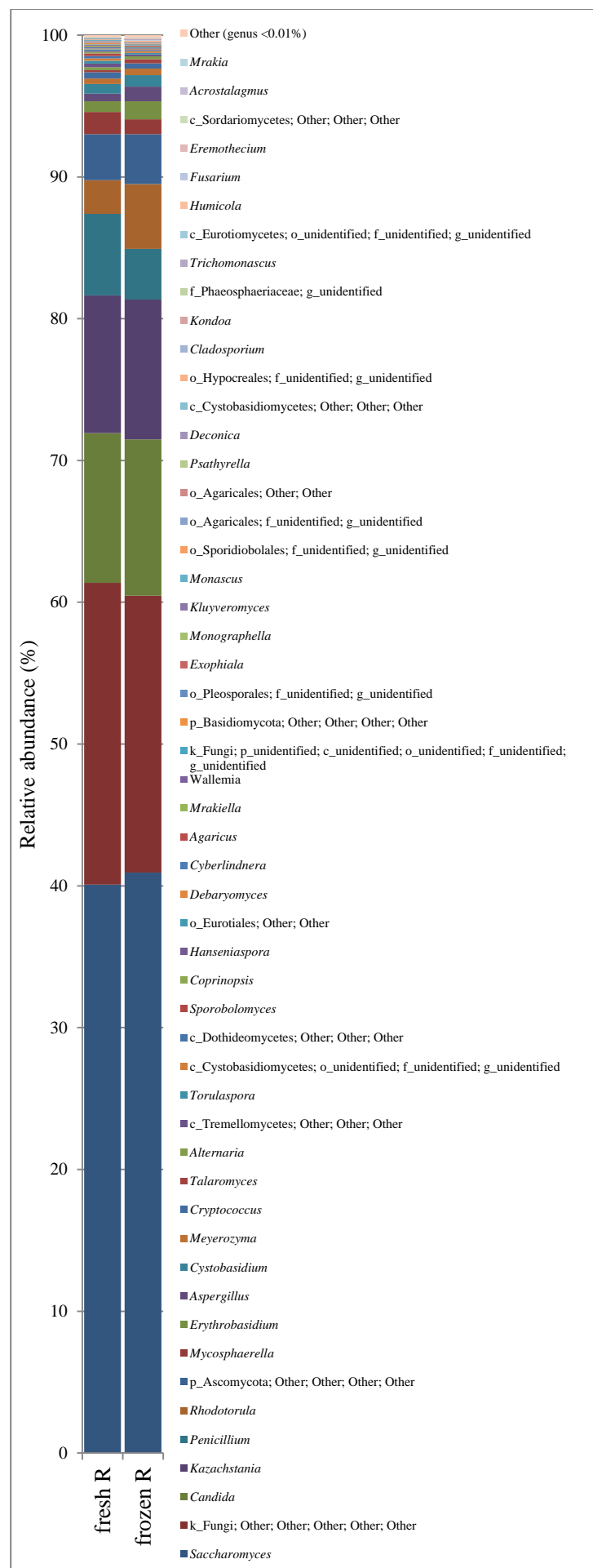

Freezing overview (genus level), genera at >0.01% (A) BB freezing (B) R freezing;

Supplement: Supplementary file 5 [file Image_3.PDF]
